# Frequency response areas of neurons in the mouse inferior colliculus. III. Time-domain responses: Constancy, dynamics, and precision in relation to spectral resolution, and perception in the time domain

**DOI:** 10.1371/journal.pone.0240853

**Published:** 2020-10-26

**Authors:** Marina A. Egorova, Alexander G. Akimov, Gleb D. Khorunzhii, Günter Ehret

**Affiliations:** 1 Sechenov Institute of Evolutionary Physiology and Biochemistry, Russian Academy of Sciences, St. Petersburg, Russia; 2 Institute of Neurobiology, University of Ulm, Ulm, Germany; Universidad de Salamanca, SPAIN

## Abstract

The auditory midbrain (central nucleus of inferior colliculus, ICC) receives multiple brainstem projections and recodes auditory information for perception in higher centers. Many neural response characteristics are represented in gradients (maps) in the three-dimensional ICC space. Map overlap suggests that neurons, depending on their ICC location, encode information in several domains simultaneously by different aspects of their responses. Thus, interdependence of coding, e.g. in spectral and temporal domains, seems to be a general ICC principle. Studies on covariation of response properties and possible impact on sound perception are, however, rare. Here, we evaluated tone-evoked single neuron activity from the mouse ICC and compared shapes of excitatory frequency-response areas (including strength and shape of inhibition within and around the excitatory area; classes I, II, III) with types of temporal response patterns and first-spike response latencies. Analyses showed covariation of sharpness of frequency tuning with constancy and precision of responding to tone onsets. Highest precision (first-spike latency jitter < 1 ms) and stable *phasic* responses throughout frequency-response areas were the quality mainly of class III neurons with broad frequency tuning, least influenced by inhibition. Class II neurons with narrow frequency tuning and dominating inhibitory influence were unsuitable for time domain coding with high precision. The ICC center seems specialized rather for high spectral resolution (class II presence), lateral parts for constantly precise responding to sound onsets (class III presence). Further, the variation of tone-response latencies in the frequency-response areas of individual neurons with *phasic*, *tonic*, *phasic-tonic*, or *pauser* responses gave rise to the definition of a core area, which represented a time window of about 20 ms from tone onset for tone-onset responding of the whole ICC. This time window corresponds to the roughly 20 ms shortest time interval that was found critical in several auditory perceptual tasks in humans and mice.

## Introduction

Perceiving and responding to complex sounds such as speech, music, and animal calls requires analyses of sound patterns in the spectral and temporal domains. The neural codes in both domains are generally uniform in auditory nerve fibers and diversify greatly in higher centers of the auditory pathways [1, 2]. In the central nucleus of the midbrain inferior colliculus (ICC), which is in the focus of our present study, 11 major ascending pathways with preprocessed information from auditory brainstem centers converge [3–9]. This input convergence creates, together with intrinsic properties of local neurons and excitatory and inhibitory local microcircuits a huge diversity of neuronal response properties [10–17]. Examples of such properties are various spontaneous and stimulus-driven spike rates, temporal response patterns, and tone-response latencies within neurons’ frequency-response areas (FRAs) which, by themselves, are of various shapes [5, 7, 18–30].

Descriptions of systematic distributions of many neuronal response properties along certain coordinates in the three-dimensional ICC space provide aspects of both order in the diversities of responses and, likely, functional relationships to auditory perceptual abilities and acuities. Besides the definition of frequency-band laminae by small gradients of characteristic frequencies (CFs) of neurons within layers orthogonal to the main tonotopic axis [31], nine spatial gradients of values of neural response parameters including the general tonotopy (expressed by major changes of CFs of the neurons) have been identified [32]. They relate to tone-response threshold [33–35], tone-response latency and latency jitter [36–39], best-modulation frequency to amplitude modulated tones (periodotopy [36, 38, 40], sharpness of frequency tuning to excitatory tones [36, 41, 42], shapes of excitatory FRAs [43, 44], temporal tone-response patterns [44], and preferred responses to velocities and directions of frequency sweeps [44]. Unless the ICC is divided in several compartments, the three spatial dimensions of the ICC cannot house nine spatial gradients of independent response parameters. Since only a single continuous main tonotopic gradient from the dorsolateral to the ventromedial ICC has been found in the mammalian ICC [5, 7, 31, 45], the ICC appears to be just one unit and, accordingly, demands interdependence or covariation of spatial gradients of some neuronal response parameters.

The first objective of the present study, therefore, is to analyze possible covariation of neuronal response parameters in the spectral and temporal domains. Specifically, we compare shapes of excitatory FRAs (including sharpness of tuning, presence and strength of inhibitory areas, and bandwidths of frequency resolution) of neurons with their temporal response patterns, tone-response latencies, and the precision of tone-onset coding (latency jitter). The hypothesis is that ICC neurons specialize in spectral or temporal coding as has been suggested using a different experimental approach, i.e. recordings of spectrotemporal receptive fields with frequency/amplitude-modulated sounds in the ICC of the cat [46, 47]. Also, the kind and degree of specialization may bear reference to their location in the ICC.

The second objective concerns the possible relationship between response stability within the FRA and constancy of perception. The hypothesis is that the constancy of perception and sound-discrimination ability reached by sound levels of about 30 dB above hearing threshold (e.g. speech intelligibility [48], temporal gap detection in humans and animals [49–51], vowel second formant discrimination in cats [52], minimum perceptual frequency difference of tones or intensity difference of noise in humans [53]) is reflected by above-threshold stability of temporal response parameters in the neurons’ FRAs.

Our results show covariation of FRA shape with phasic onset-responses to tones, with the precision (jitter) of onset responding, and with a possible neuronal position in the lateral ICC. The variation of first-spike latencies in neurons with short-latency responses is the basis for defining a core area within the FRA which provides conditions necessary for supporting perceptual constancy important, for example, in the perception of mouse communication sounds and phonemes of human speech.

## Materials and methods

The present study on temporal response characteristics of ICC neurons is based on previous recordings that had been evaluated with regard to excitatory and inhibitory tuning characteristics before [23]. In addition to the neurons which served as data base in the Egorova et al. study [23], the present study also includes 13 neurons of which only excitatory frequency response areas had been recorded before. The evaluation of the precision of tone-response latencies in the present study is based on published recordings of responses from excitatory FRAs of ICC neurons [43, 54–56]. Since detailed descriptions of materials and methods have already been published [23, 54], only some essential points are mentioned here.

### Subjects and surgery

Virgin female house mice (Mus musculus, hybrids of outbred strain NMRI and feral mice [23, 43]; F1 hybrids of CBA and C57BL/6 strain [43, 54–56]) aged 8–15 weeks, were anesthetized (0.2% oxygen, 1.5–1.8% halothane [23, 43]; 120 mg/kg body weight ketamine plus 5 mg/kg xylazine intraperitoneally [43, 54–56]) to perform a craniotomy over the left side IC. The dura overlaying the IC was removed and warm silicon oil applied to the brain surface. The anesthetic state (animal quiet without indication of pain or distress, but presence of weak reflex reactions to foot or tail pinching) was maintained by intraperitoneal injection of ketamine (35 mg⁄kg) and xylazine (1.0 mg⁄kg) about every 20–45 min. The animal’s rectal temperature was kept at 37 ± 1° C by a feedback-controlled heating pad. At the end of the experiment, the anesthetized animal was killed by cervical dislocation [23] or by an overdose of anesthetic [54–56]. The experiments were carried out in accordance with the European Communities Council Directives (86/609/EEC and 2010/63EU) and were approved by the Regierungspräsidium Tübingen (Germany) or the Russian Foundation for Basic Research.

### Electrophysiological recording

The recording electrodes (glass pipettes filled with 3 M KCl, impedances 4–8 MΩ) were placed stereotaxically in an area comprising the mouse ICC (1.0–1.5 mm caudal and 0.8–1.5 mm lateral of the λ-point [33, 57, 58]. Neural responses were amplified 10.000 times, bandpass-filtered (0.3–10 kHz) and fed in parallel to an oscilloscope, audiomonitor and window discriminator, whose output was fed to a computer for analysis and storage of the spike data together with the timing of the tone stimuli.

### Stimulus generation

Tone bursts were generated either by a computer controlled Tacita [23] or TMS 320 C30 [43, 54–56] D/A converter system. They were initiated at zero phase, had a duration of 60 ms [23, 43] or 100 ms [54–56] including 5 ms trapezoidal rise and fall times in both cases, and were delivered at intervals of 300 ms [23, 43] or 800 ms [54–56]. With additional intensity adjustment via dB attenuators and amplifiers for the loudspeakers, the total ranges of sound pressure levels (SPLs) available without significant harmonic distortion products were from -20 dB to 90 dB [23] or -10 dB to 100 dB [43, 54–56]. SPLs of the tone bursts were measured and calibrated at the right pinna of the experimental animal (Bruel and Kjaer microphone 4135, preamplifier 2633, measuring amplifier 2606 or 2636). The sound spectrum in the range of 1–75 kHz [23] or 1–50 kHz [54–56] was flat with +/- 1.5 dB or +/- 6 dB variation, respectively. Tones were presented free-field from anterior, 45° to the right of the mouse sagittal plane and thus contralateral to the recorded ICC.

### Experimental procedures

Recordings were done in a sound-proof and anechoic room. Tone bursts of variable frequencies and intensities (frequency generator, electronic switch, dB attenuator, speaker-amplifier, loudspeaker) were used as search stimuli. When a single unit was isolated (spikes of defined shape and amplitude were triggered by the tone bursts and observed at an oscilloscope), its excitatory CF and lowest excitatory threshold (the threshold at CF) were determined audiovisually. In this way, the following automatic computer-controlled measurement of the neuron’s excitatory FRA was centered at the approximate CF. The measurement of the FRA was performed by presenting pseudo-random sequences of 16 x 16 = 256 [23] or 45 x 15 = 675 [43, 54–56] different frequency-intensity combinations covering a frequency range from about 2 octaves below to 1 octave above the estimated CF. Each frequency-intensity combination was presented 3 times [23] or once [43, 54–56]. Steps between levels were 5–7 dB [23] or 5 dB [43, 54–56]. Frequency steps were equidistant on a logarithmic frequency scale. After the FRA was measured, the exact CF and the absolute threshold at the CF were determined. The recorded data were used for offline analysis of the neurons’ tone-response patterns and latencies. The original recordings [23, 54] included two-tone stimulation paradigms measuring areas of inhibition (and the strength of inhibition) within and around the excitatory FRA of a given neuron. Thus, the absence/presence of inhibition as a characteristic of neurons in the three classes of neurons (see Results) could be considered in the discussion of the results.

### Data analyses

**Fig 1:**
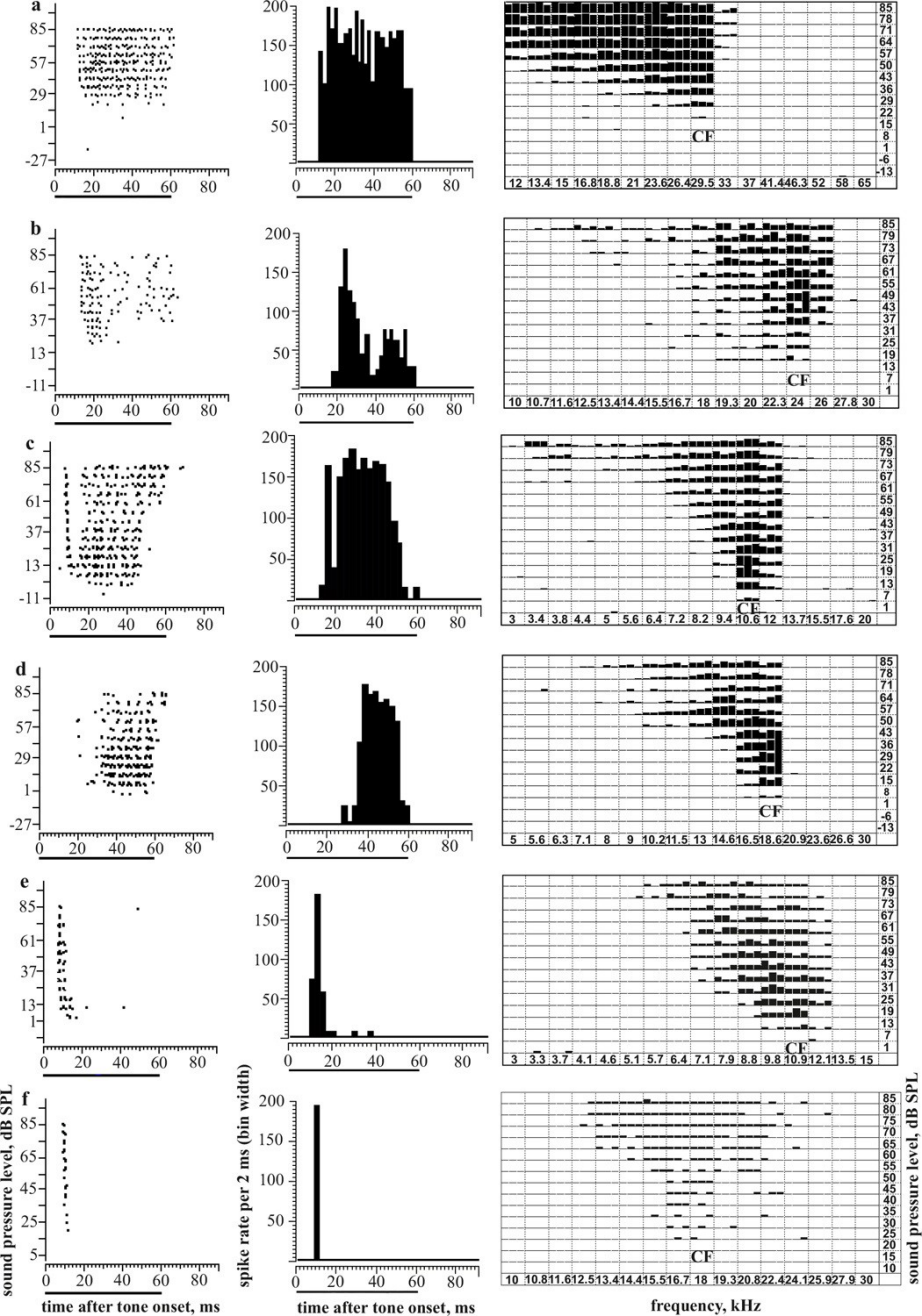
Neural response analyses in the time and frequency domains. Examples of spike responses (raster plots; left) of ICC neurons to tones at the characteristic frequency (CF), respective PSTHs taken 30 dB above threshold at CF (middle), and responses (the number of spikes of three responses per frequency-intensity dyad) defining the excitatory frequency response area with the CF indicated (right). Solid horizontal lines below the x-axes (left, middle) indicate tone duration. The binwidth of the PSTHs is 2 ms. Examples of different temporal response patterns are shown. a: *tonic*; b: *phasic-tonic*; c: *pauser*; d: *long-latency*; e, f: *phasic*. Neurons in a–e have shapes of frequency-response areas of class I, the neuron in f of class III.

**Fig 2:**
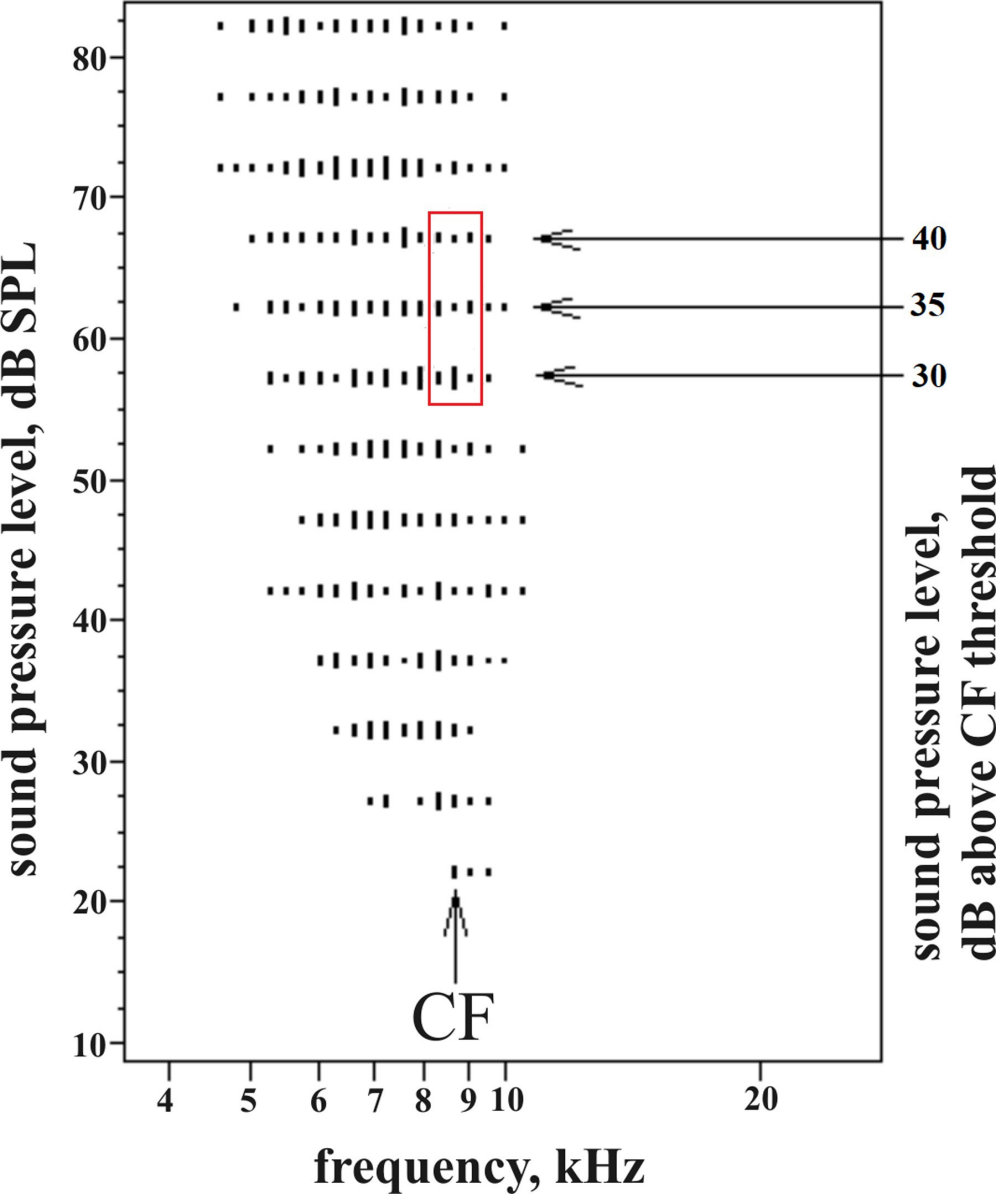
Analysis of the precision of tone-onset response latency. For every neuron, the first-spike latencies of the 9 responses framed by the red box were measured. These responses always concerned the CF- tone and the next lower and higher frequencies at tone levels 30, 35 and 40 dB above CF threshold (arrows). The excitatory frequency response area (example shows a class II neuron) is defined by the vertical dashes (number of spikes to a single tone burst).

Temporal response characteristics of neurons were evaluated via raster plots and peristimulus-time histograms (PSTHs) at frequency-intensity combinations in the neurons’ whole FRAs (Fig 1). Latencies of responses in the FRA [23] were determined as the first-spike latency at a given frequency-intensity combination, measured with +/-1 ms precision, and averaged from the measurements of the three repetitions of the same stimulus (see FRAs in Fig 1). The precision of the latency of a neuron to tone onset was determined by the standard deviation (SD) of the average of the 9 latencies 30 dB, 35 dB, and 40 dB above CF threshold at the CF and at the next lower and higher frequencies in the neuron’s FRA [43, 54–56] (see Fig 2). In this case, single latencies were determined with a resolution of +/- 10 μs. All latency values in this study are given after subtraction of the run time of the sound from the loudspeakers to the animal’s ear.

The statistical tests applied (ANOVA, ANOVA on ranks, U-test, Chi^2^-test, F-test, linear regression) were two-tailed with α = 0.05. Chi^2^-tests were always calculated with numbers of neurons not with percentages of distributions of neurons. In cases where the ANOVA on ranks indicated significant differences, the U-test (with Bonferroni correction for multiple testing, if applicable) was applied to assess the final p-value. In addition, the Shapiro-Wilk test (α = 0.05) was used to check for normality of a data sample, and the test of Dixon [59] was used to identify outliers (α = 0.05). Statistics, except the outlier test and the F-test [60] were run with SigmaPlot software (version 11).

## Results

### General characteristics

The results were based on a sample of 135 single ICC units from 45 mice with CFs between 4.3 and 60 kHz (tone response threshold between -20–53 dB SPL) [23] and on 178 single ICC units with CFs between 4.5 and 42.2 kHz (tone response thresholds between -10–50 dB SPL) recorded in 68 mice [43, 54–56]. The shapes of the FRAs of the neurons were classified as we did before [23] and also done by others [28, 61] in class I with a steep slope (> 250 dB/octave) on the high-frequency side and a shallow slope (< 150 dB/octave) on the low-frequency side of the FRA (examples are in Figs 1A–1E and S1A and S1B, S3A, S4A, S5A and S5B and S6C), in class II with steep slopes (low-frequency slope > 150 dB/octave; high-frequency slope > 250 dB/octave) on both sides (examples are in Figs 2 and S2A and S2B and S6B), and in class III with shallow slopes (low-frequency slope < 150 dB/octave; high-frequency slope < 250 dB/octave) on both sides (examples are in Figs 1F and S3B, S4B, S5C and S6A). The differences in the shapes of FRAs of the classified neurons were also reflected by conventional Q-values expressing the widths of FRAs. The average Q_30_ (Q_40_) values (CF divided by the FRA bandwidth at 30 dB (Q_30_) or 40 dB (Q_40_) above threshold) with standard deviations of the neurons in the three classes were the following: class I: 3.431 ± 1.937 (2.787 ± 1.443), class II: 7.278 ± 8.235 (6.400 ± 8.655), class III: 2.382 ± 1.975 (1.676 ± 0.986). In each case, the Q-values differed significantly among the classes (ANOVA on ranks, p < 0.001 in each case) with significant differences between the classes (U-tests p < 0.01 for Q_30_; p < 0.001 for Q_40_ class III vs. class I and class II). On average therefore, class II neurons had the sharpest excitatory frequency tuning, followed by neurons of class I. Class III neurons had the average widest FRAs. We did not include class IV neurons with two CFs [23] in our present analysis because their small number (8 neurons) in our sample hampered meaningful statistical comparisons.

**Fig 3:**
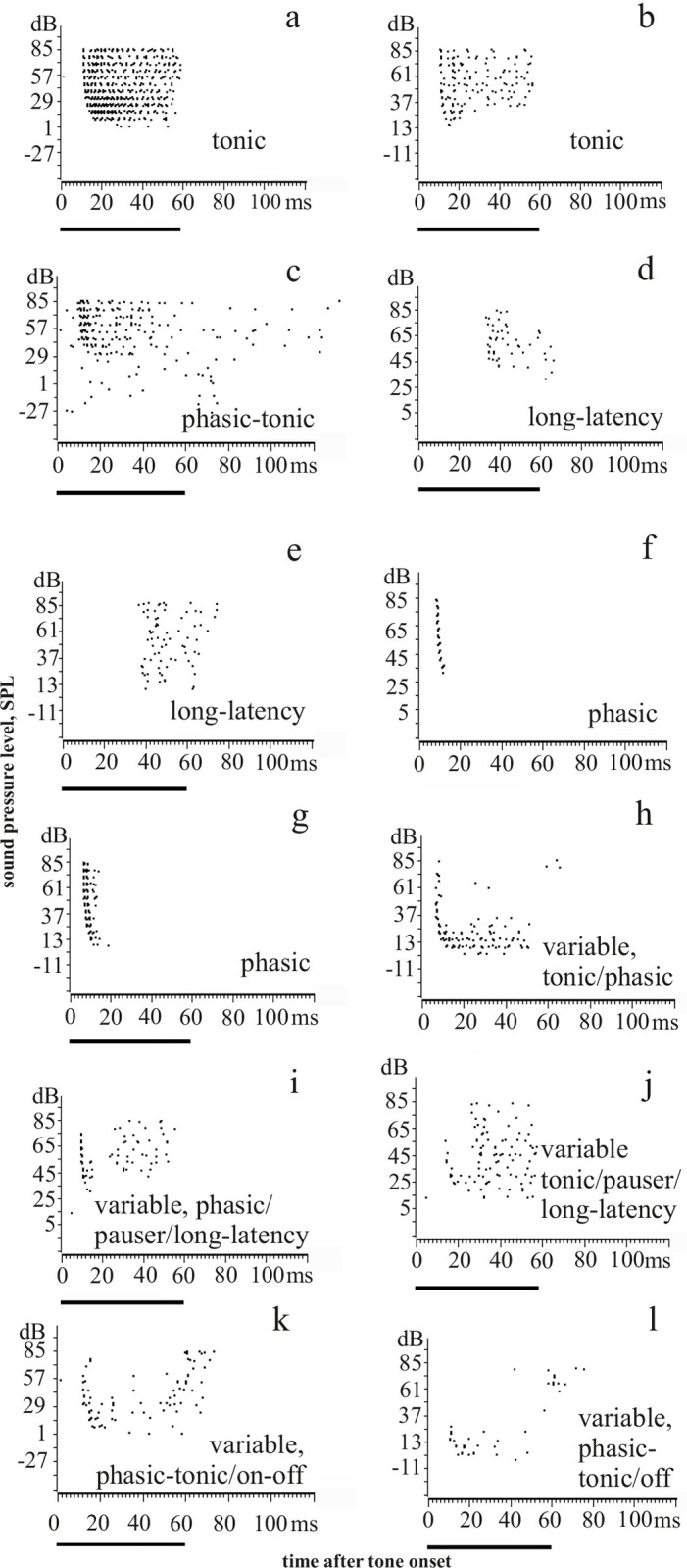
Examples of spike responses (raster plots) of ICC neurons to tones at the characteristic frequency. Panels a–g show constant patterns (no change of pattern with tone level), panels h–l show variable patterns. The type of pattern is indicated in each panel. Solid horizontal lines below the x-axes indicate tone duration.

The temporal response characteristics derived from raster plots and PSTHs were classified as *tonic* (spikes during tone duration without prominent peak(s), Figs 1A and 3A), *phasic-tonic* (short-latency response peak followed by a tonic response at a lower rate throughout tone duration, Figs 1B and 3B), *pauser* (short-latency response peak followed by a pause with no or few spikes and a tonic response, Fig 1C and, in part Fig 3I), *phasic* (short-latency response peak to tone onset that might be followed by few spikes with response ending before the end of the tone, Figs 1E and 1F and 3F and 3G), and *long-latency* or *build-up* (rather tonic response starting with a latency of more than about 20 ms after tone onset, Figs 1D and 3D and 3E). Further examples of the defined temporal response patterns can be found in the figures of the supporting information, *tonic* (S1A Fig), *phasic* (S1B and S2A and S2B Figs), *phasic-tonic* (S3A Fig at CF; S4A Fig at low frequencies and levels), *pauser* (S3B Fig at CF; S4B Fig at CF; S5A and S5C Fig for frequencies at and near CF), *long-latency* (S5A and S5C Fig at low frequencies; S6A and S6B Fig). The here described tone-evoked temporal response patterns of ICC neurons have been found in the mouse ICC before [14, 16, 21, 44, 62, 63] and are common also to the ICC of other mammals [5, 10, 12, 13, 15, 17–20, 25, 35, 64].

### Temporal response characteristics at the neuron’s CF: Response patterns

**Fig 4:**
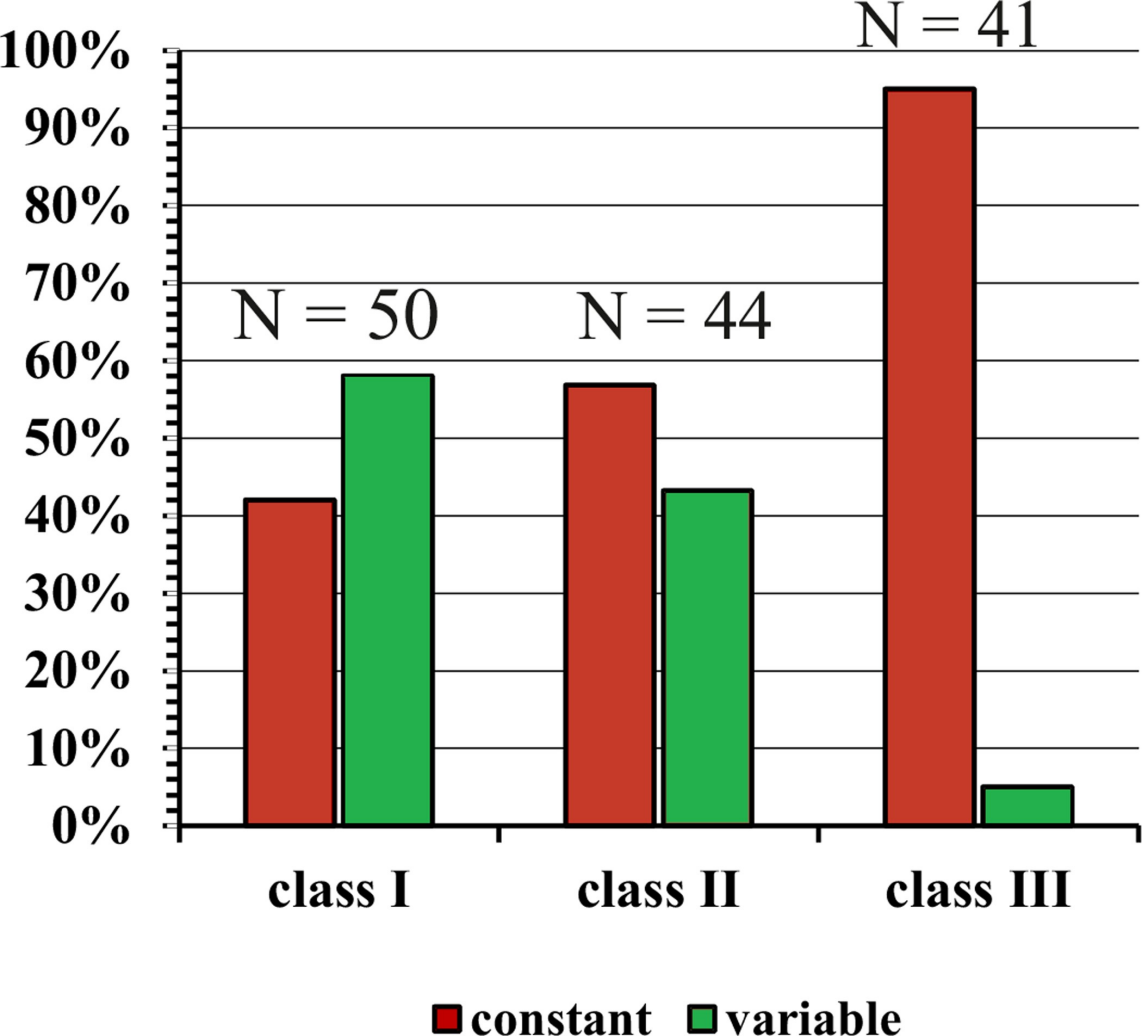
Percentages of neurons in the three classes of FRAs (class I–III) with constant (red) or variable (dark green) temporal response patterns. Constant and variable refers to CF tones of at least 30 dB above response threshold. N = numbers of neurons in the respective FRA classes.

The above defined temporal response patterns of the neurons often changed with increasing sound level at the CF. Most of such changes occurred when the neurons were stimulated by soft tones at levels of up to about 30 dB above their response threshold. Examples of changes are shown in Fig 1C, (from *tonic* to *pauser*), Fig 3C (from *tonic* to *phasic-tonic*), Fig 3H (from *tonic* to *phasic*), Fig 3J (from *tonic* to *pauser* to *long-latency*), Fig 3I (from *phasic* to *pauser* to *long-latency*), Fig 3K (from *phasic-tonic* to *phasic* and *off-response*), and Fig 3L (from *on-* to *off-response*). Fig 4 shows for the neurons in the three classes of FRAs the distribution of those which had constant temporal response patterns at levels of 30 dB and more above response threshold at CF (Figs 1A–1F and 3A–3H), and of the others which further changed their patterns at sound levels more than 30 dB above threshold. Altogether 85 neurons (63%) had constant and 50 neurons (27%) variable response patterns. Almost all class III neurons (39 of 41) had constant patterns, while the patterns changed in nearly 50% of the neurons in classes I (in 29 of 50 neurons) and II (in 19 of 44 neurons). The distributions of constant and variable temporal response patterns differed significantly between the classes (Chi^2^ = 28.3; 2 degrees of freedom; p < 0.001) mainly because most class III neurons had constant patterns.

**Fig 5:**
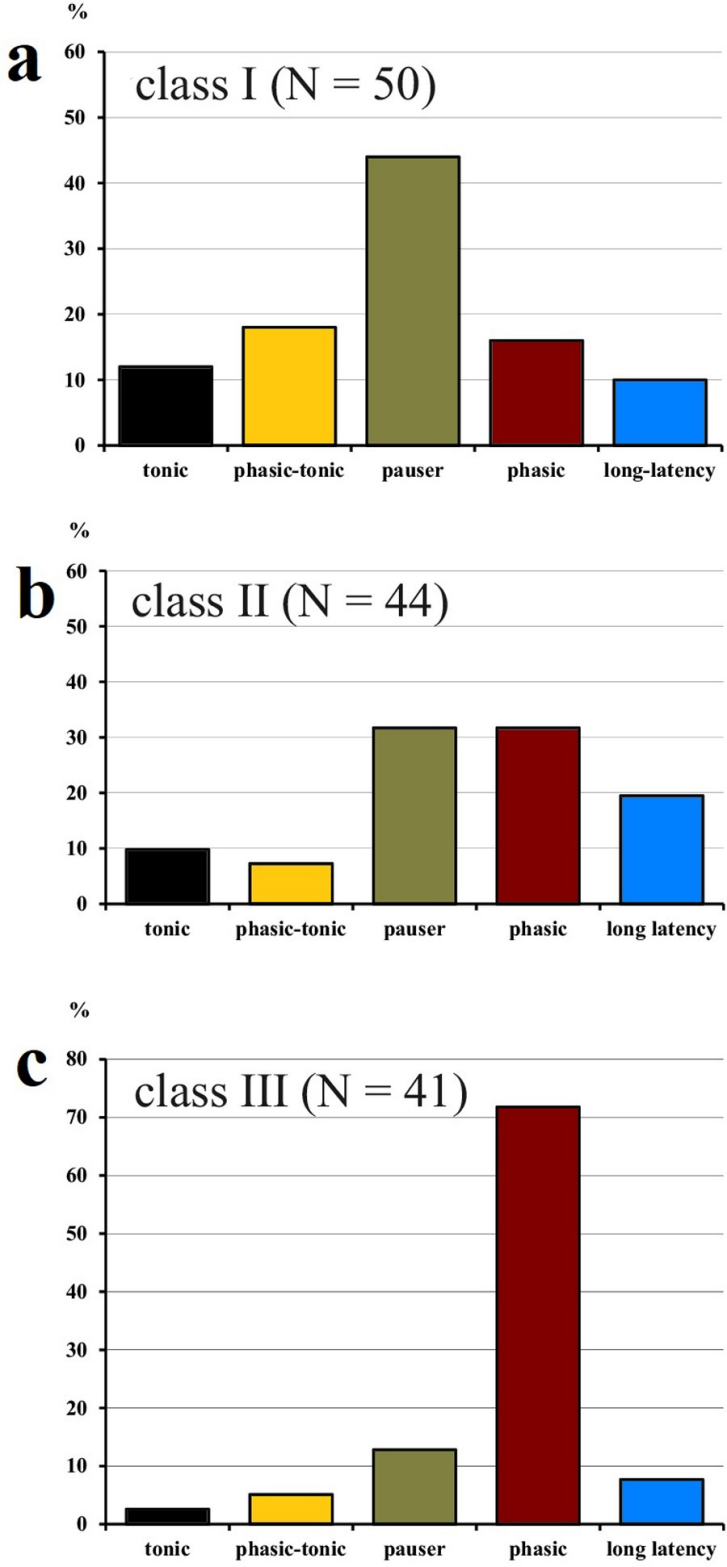
Percentages of neurons with the indicated temporal response patterns at 30 dB above CF-tone threshold. Distributions are shown separately for the neurons in the three classes of FRAs (а: class I; b: class II; c: class III).

At 30 dB above response threshold at CF, the temporal response patterns in the three classes of FRAs divided up as shown in Fig 5. The numbers of neurons with different temporal response patterns in the classes of FRAs are also given in Table 1. Neither the numbers of class I nor of class II neurons in the five classes of temporal response patterns (*tonic*, *phasic-tonic*, *pauser*, *phasic*, *long-latency*; Table 1 and Fig 5) differed significantly from an equal distribution (class I: Chi^2^ = 7.44, class II: Chi^2^ = 7.09; 5 degrees of freedom each; both non-significant). Different from neurons in classes I and II, class III neurons were specialized as *phasic* responders (Chi^2^ = 23.5; 5 degrees of freedom; p < 0.001). The distributions (Fig 5) differed significantly between the classes (Chi^2^ = 35.2; 8 degrees of freedom; p < 0.001), with the high incidence of *phasic* patterns in class III contributing most to the significant difference.

**Table 1:** Tone-response latencies at CF at 30 dB above CF threshold of neurons in the different classes of FRAs and of different temporal response patterns.

Neuron class		tonic	phasic-tonic	pauser	phasic	all short latency	long-latency
**Class I**	**mean**	**11.00**	**10.33**	**10.73**	**10.63**	**10.67**	**23.80**
	**SD**	**5.18**	**2.12**	**3.52**	**3.07**	**3.47**	**4.76**
	**range**	**4–19**	**7–13**	**7–18**	**8–17**	**4–19**	**20–30**
	**N**	**6**	**9**	**22**	**8**	**45**	**5**
**Class II**	**mean**	**12.25**	**10.33**	**12.21**	**11.07**	**11.07**	**27.00**
	**SD**	**2.99**	**1.53**	**3.64**	**2.79**	**2.74**	**6.98**
	**range**	**9–16**	**9–12**	**6–19**	**8–16**	**6–19**	**21–40**
	**N**	**4**	**3**	**14**	**14**	**35**	**9**
**Class III**	**mean**	**12.00**	**14.50**	**7.83**	**10.93**	**11.32**	**26.67**
	**SD**	**-**	**0.71**	**2.56**	**2.78**	**2.02**	**10.07**
	**range**	**-**	**14–15**	**6–13**	**7–17**	**6–17**	**20–36**
	**N**	**1**	**2**	**6**	**29**	**38**	**3**
	**mean**	**11.55**	**10.93**	**10.79**	**10.54**	**10.95**	**26.00**
**all**	**SD**	**4.06**	**2.34**	**3.59**	**2.59**	**3.15**	**6.70**
	**range**	**4–19**	**8–15**	**6–19**	**7–17**	**4–19**	**20–40**
	**N**	**11**	**14**	**42**	**51**	**118**	**17**

Average values (ms) with standard deviations (SD), ranges of latencies (ms) and the number of neurons (N) in the different response groups are shown.

**Fig 6:**
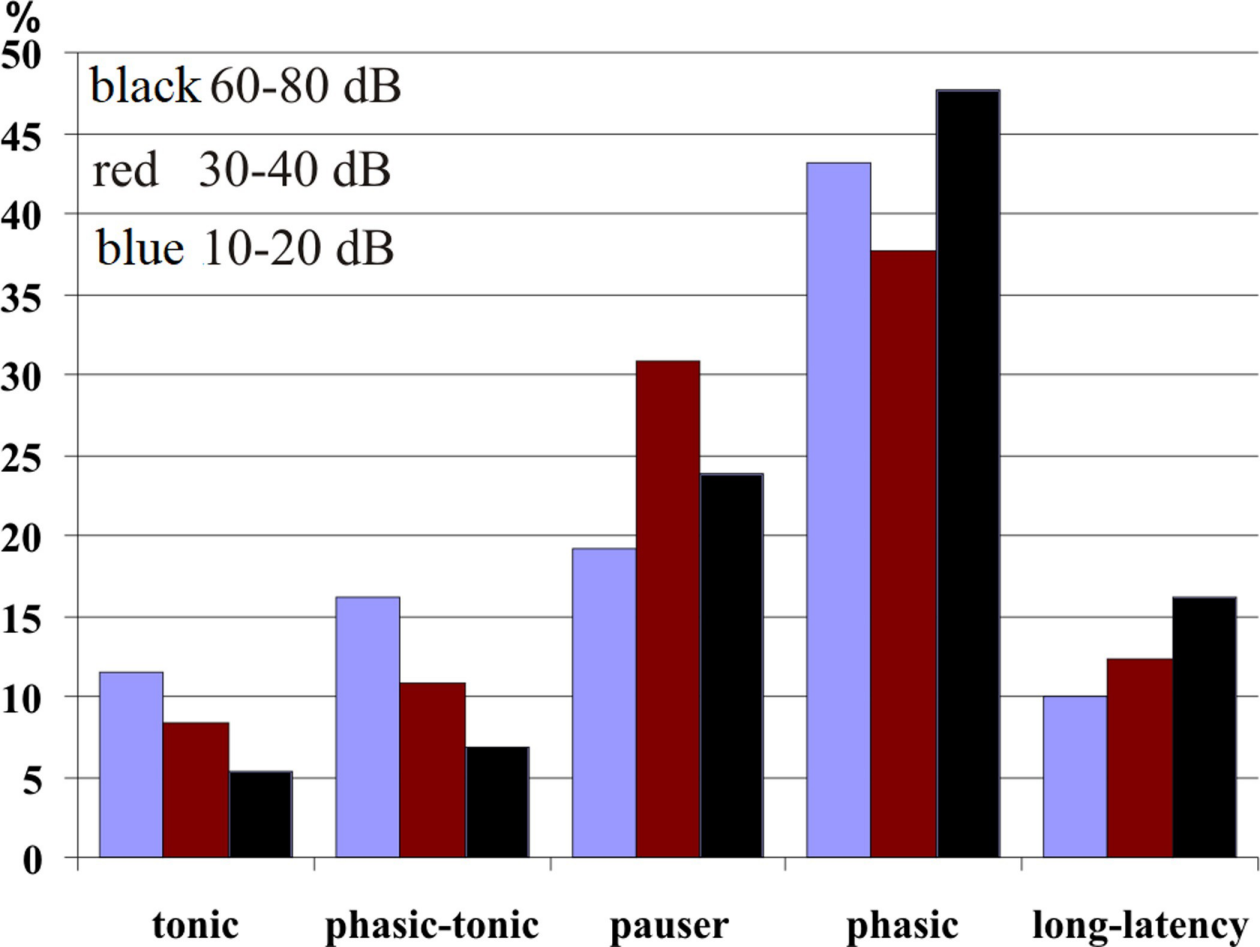
Percentages of neurons (N = 50) which changed their temporal response patterns depending on the tone level at CF. Of the 50 neurons, 48 had FRAs of classes I and II. Three ranges of tone levels above CF threshold are indicated: 10–20 dB (blue); 30–40 dB (red), 60–80 dB (black).

Fig 4 showed that many class I and II but only very few class III neurons changed their temporal response patterns at higher sound levels. For these 50 neurons (37% of our sample) the development of the incidences of temporal response patterns for three ranges of level above CF threshold (10–20 dB, 30–40 dB, 60–80 dB) is shown in Fig 6. With increasing level, *tonic* and *phasic-tonic* patterns decreased while long*-latency* patterns increased. The incidences of *pauser* and *phasic* responses varied and were about 4% higher at 60–80 dB than at 10–20 dB above threshold.

In summary, Figs 4 and 5 indicate that neurons with class III FRAs preferentially responded with *phasic* patterns that were constant at sound levels of 30 dB or more above the neurons’ response thresholds at CF. Temporal response patterns of nearly 50% of class I and II neurons were more variable (Figs 4 and 5) and changed with sound level (Fig 6).

### Temporal response characteristics at the neuron’s CF: Response latencies

**Fig 7:**
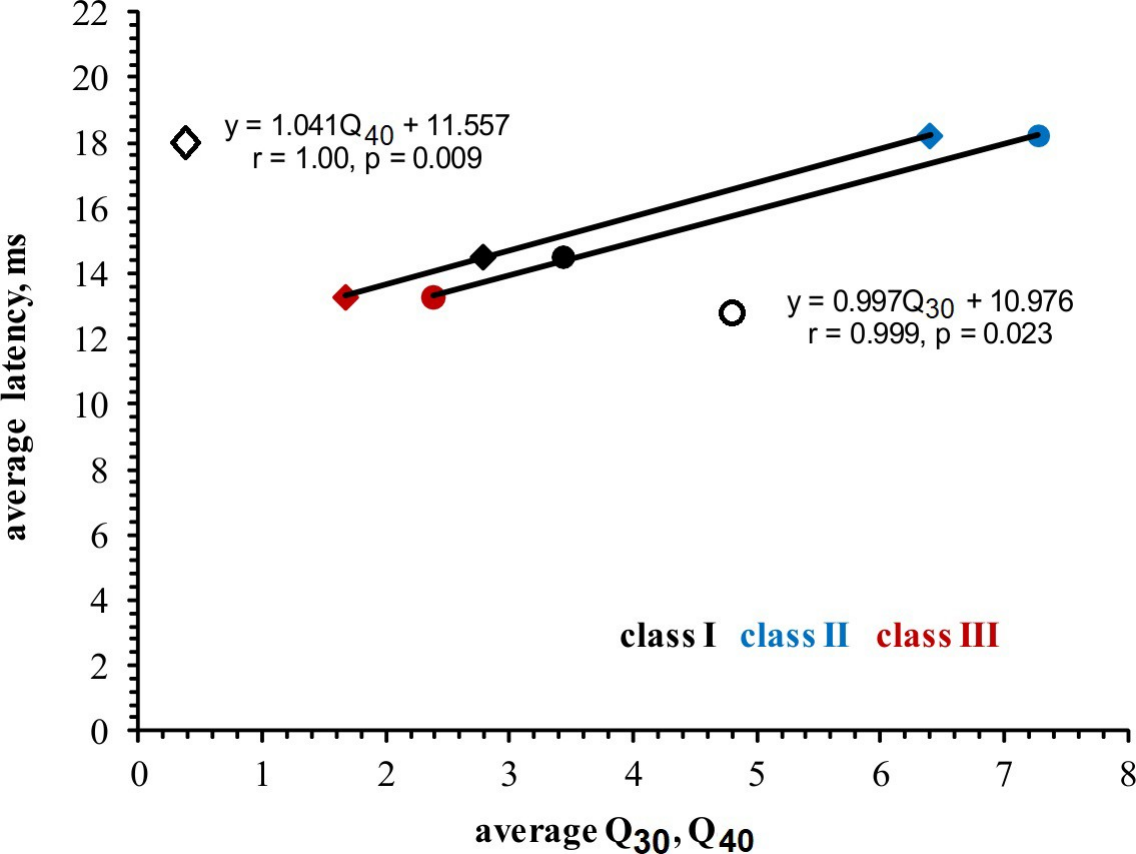
Relationship between average first-spike latencies of the neurons in the three classes and the average widths of their FRAs (expressed as Q_30_ or Q_40_ values). The statistically significant linear regressions (see equations, r and p values) show that, on a global scale, latencies increase with sharpening of excitatory frequency tuning (increase of Q_30_ and Q_40_).

In general, tone-response latencies varied widely in the neurons’ FRAs. When we calculated the average first-spike latency from all responses in a neuron’s FRA and averaged these latencies separately for the neurons in the three classes, we got the following values (ms): class I: 14.50 ± 5.88; class II: 18.21 ± 9.29; class III: 13.27 ± 6.30. These values differed among the classes (ANOVA on ranks, p < 0.005). Class II neurons had significantly longer average whole FRA latencies than class I and class III neurons (U-test, p < 0.05, p < 0.001, respectively). In general, the first-spike latencies correlated significantly (linear regressions) with the widths of the FRAs expressed by the Q_30_ and Q_40_ values as shown for all neurons in S7A and S7B Fig. This means that, on average, first-spike latencies increased with increasing sharpness of frequency tuning. This relation was even more obvious when we plotted the average first-spike latencies of the classes versus the respective average Q_30_ and Q_40_ values of the classes (Fig 7). We noted significant regression lines with slopes very close to 1.00 (Fig 7), i.e. class III with shortest latencies and widest FRAs, class I with intermediate latencies and intermediate width of FRAs and class II with longest latencies and narrow FRAs.

In further latency analyses, we looked at details of latency changes within the FRAs of neurons and at relationships between latencies and temporal response patterns. In general, first-spike latencies depend on the tone level and the rise time of the tone level at tone onset [65, 66]. Latencies are longest near response threshold of the neurons and with long rise times of the tones. At about 30 dB above threshold, rather constantly short latencies were reached with rise times of about 5 ms and shorter [65, 66]. Since our tone bursts had rise times of 5 ms, we determined tone-response latencies at 30 dB above tone-response threshold in order to have a common, on average rather level-independent, basis for latency comparisons between neurons of different temporal-response patterns and different shapes of FRAs. An average constancy of tone-response latencies for sound levels of 30 dB and more above response threshold had been found in neurons of the bat and mouse ICC before [24, 67].

**Fig 8:**
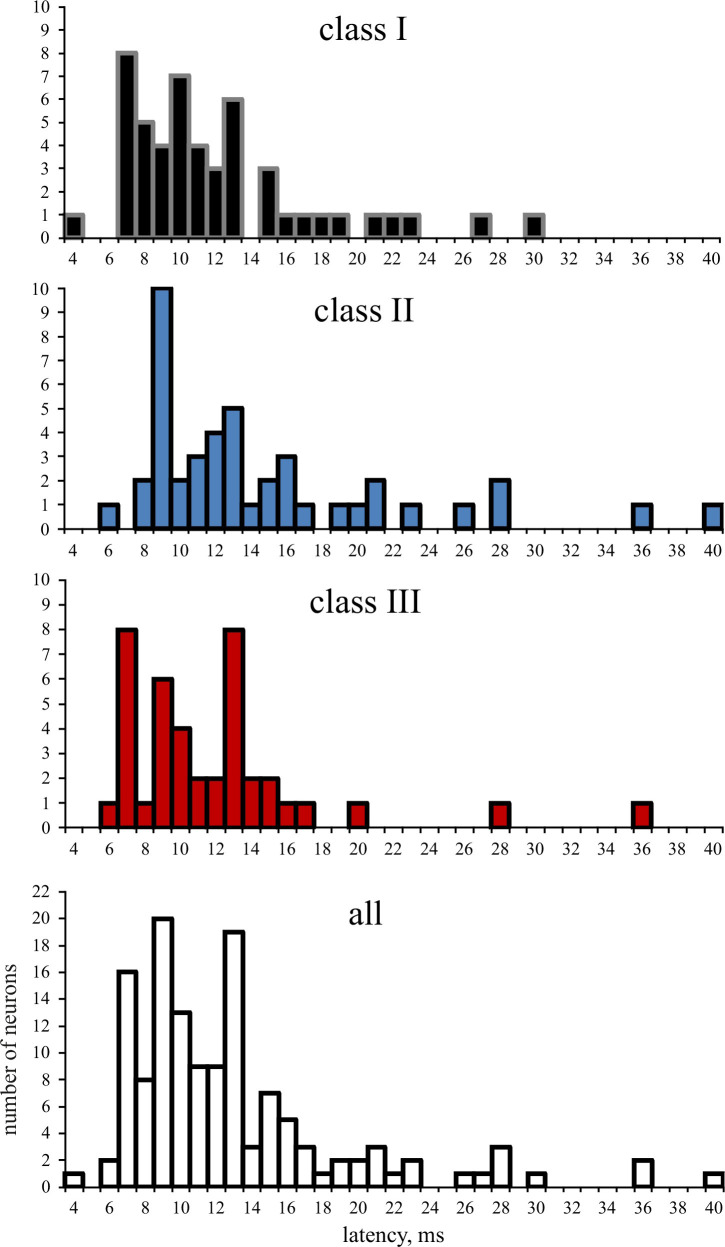
Distribution of the number of neurons with various first-spike latencies in response to CF tones at 30 dB above threshold. Latency distributions are shown separately for neurons of FRA classes I (a), II (b), III (c), and for all 135 studied neurons (d).

CF tone-response latencies at 30 dB above threshold varied widely among the recorded neurons (Fig 8). Table 1 shows the average latencies separately for the three classes of FRAs and the 5 temporal response patterns (*tonic*, *phasic-tonic*, *pauser*, *phasic*, *long-latency*). Combining *tonic* and *phasic-tonic* responses, latencies of neurons with *tonic* or *phasic-tonic* responses did not differ significantly from latencies of neurons with *pauser* or *phasic* responses in any of the 3 classes of FRAs (ANOVA with p > 0.1 in each case). Therefore, latencies from all neurons with short-latency response patterns (*tonic*, *phasic-tonic*, *pauser*, *phasic*) have been combined and are shown separately for each class of FRA (all short latency, Table 1). In tests (ANOVA) of the latencies of the short latency neurons and, separately, of the long-latency neurons for significant differences between the FRA classes (Table 1), no such differences occurred (p > 0.1 in both cases).

In summary, average tone response latencies at 30 dB above threshold at CF did neither differ among the neurons from the 3 classes of FRAs (Fig 8) nor among neurons with different short-latency temporal response patterns within each class.

### Temporal response characteristics throughout the whole excitatory receptive fields of the neurons: Defining core and peripheral parts

**Fig 9:**
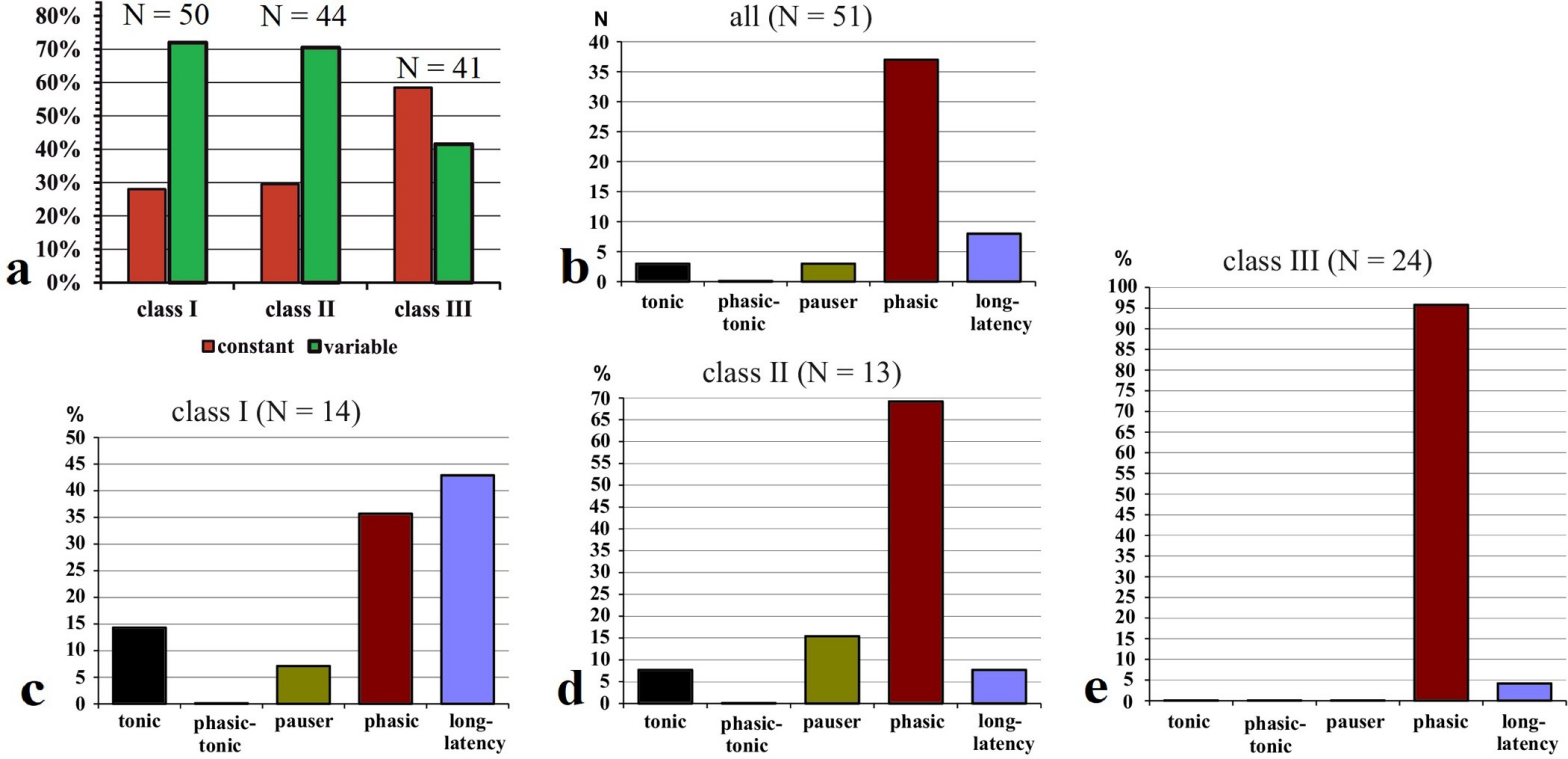
Temporal response patterns in the whole FRAs of the neurons. (a) Percentages of neurons in the three classes of FRAs (class I–III) with constant (red) or variable (dark green) temporal response patterns; (b) the distribution of 51 neurons with constant response patterns in different temporal response types; (c)–(e) percentages of neurons in a given FRA class (c: class I, d: class II, e: class III) with constant response patterns of the indicated types.

In Fig 4, we showed the distribution of neurons in the FRA classes having either constant or variable temporal response patterns at their CFs at tone levels 30 dB or more above threshold. Fig 8A shows this distribution with reference to the whole FRA. Of all 135 neurons, 51 (38%) had constant patterns in the whole FRA. The distributions of constant and variable response patterns (Fig 9A) were significantly different (Chi^2^ = 10.88; 2 degrees of freedom; p < 0.01) between the neurons of the FRA classes. Only about 30% of the neurons of classes I and II had constant patterns while nearly 60% of class III neurons had constant patterns. When we looked at the distribution of constant temporal response patterns in the FRAs of all neurons (Fig 9B), the found distribution differed significantly from a predicted equal distribution of patterns (Chi^2^-test = 39.5; 4 degrees of freedom; p < 0.001). That is, if constant temporal response patterns within the FRAs of ICC neurons were observed at all, they were *phasic* with high probability (37 of 51 = 72.5%). Examples from the 3 classes of FRAs are shown in S1 and S2 Figs. Most of these constant response patterns were *long-latency* and *phasic* (class I; Fig 9C) or *phasic* (classes II, III; Fig 9D and 9E). The distributions of the patterns of constant temporal responses (Fig 9C–9E) differed significantly between the classes of FRAs (*tonic*, *phasic-tonic*, and *pauser* responses combined; Chi^2^ = 19.7; 4 degrees of freedom; p < 0.001) mainly because class I neurons had not only constant *phasic* but also constant *long-latency* responses.

**Fig 10:**
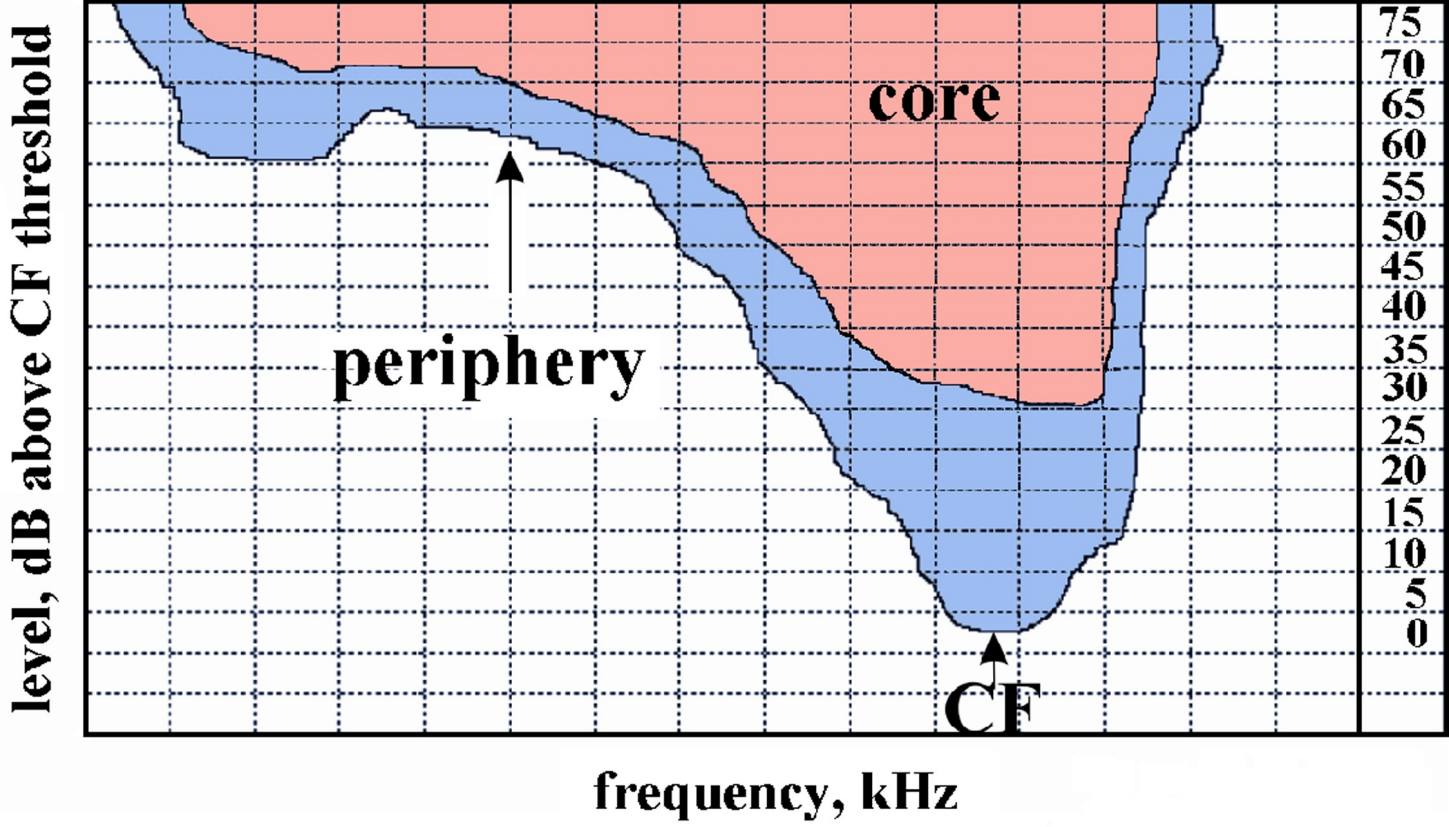
Division of the excitatory frequency response area of ICC neurons in core (pink) and periphery (blue). For definition of core and periphery, see text.

Next, we looked for neurons with constant latencies in their FRAs with reference to the latency at CF 30 dB above threshold. We found neurons with various amounts of changes of tone response latencies within their FRAs, once a certain level above threshold was reached. Neurons divided in two groups, neurons with only *phasic*, *phasic-tonic*, *pauser* or *tonic* responses and neurons with all kinds of responses, including also *long-latency* responses, within their whole FRAs. We found 89 neurons, which responded without long delay to tone onset (*phasic*, *phasic-tonic*, *pauser*, *tonic*) at all measured frequency-intensity combinations within their FRAs, that had small (+/-1 ms) up to a maximum of +/-7 ms latency differences with reference to their respective CF latency 30 dB above threshold. Larger latency deviations may have occurred only at few frequency-intensity combinations at the margins of the FRA. Examples are shown in S1–S4 Figs. The other 46 neurons with *long-latency* responses at any frequency-intensity combination within their FRAs showed latency variations of more than +/-7 ms up to +/- 37 ms. The large variations occurred at many frequency-intensity combinations at the margins of the FRAs (S5 Fig) and also at and near the CF (S6 Fig). They were caused by the loss of *phasic* components in the response or by the variability of the response latency in pure *long-latency* neurons. Therefore, we defined the core of the FRA of a neuron as the receptive field starting 30 dB above threshold at CF and extending from the CF to lower and higher frequencies with a maximum variation of tone-response latencies of +/- 7 ms for all tone levels. Peripheral parts of the response area were then the areas at and close to the CF with tone levels up to about 30 dB above threshold and the response areas at the margins of the FRAs where the latency variation was larger than +/- 7 ms with regard to the latency at CF 30 dB above threshold. This division of the whole FRA in core and peripheral areas is outlined in Fig 10. As mentioned above, FRAs of neurons responding without long delay to tone onsets (*phasic*, *phasic-tonic*, *pauser*, *tonic*) may be without a peripheral part (except near the CF), while FRAs of neurons with only *long-latency* response patterns may not have a core area.

**Fig 11:**
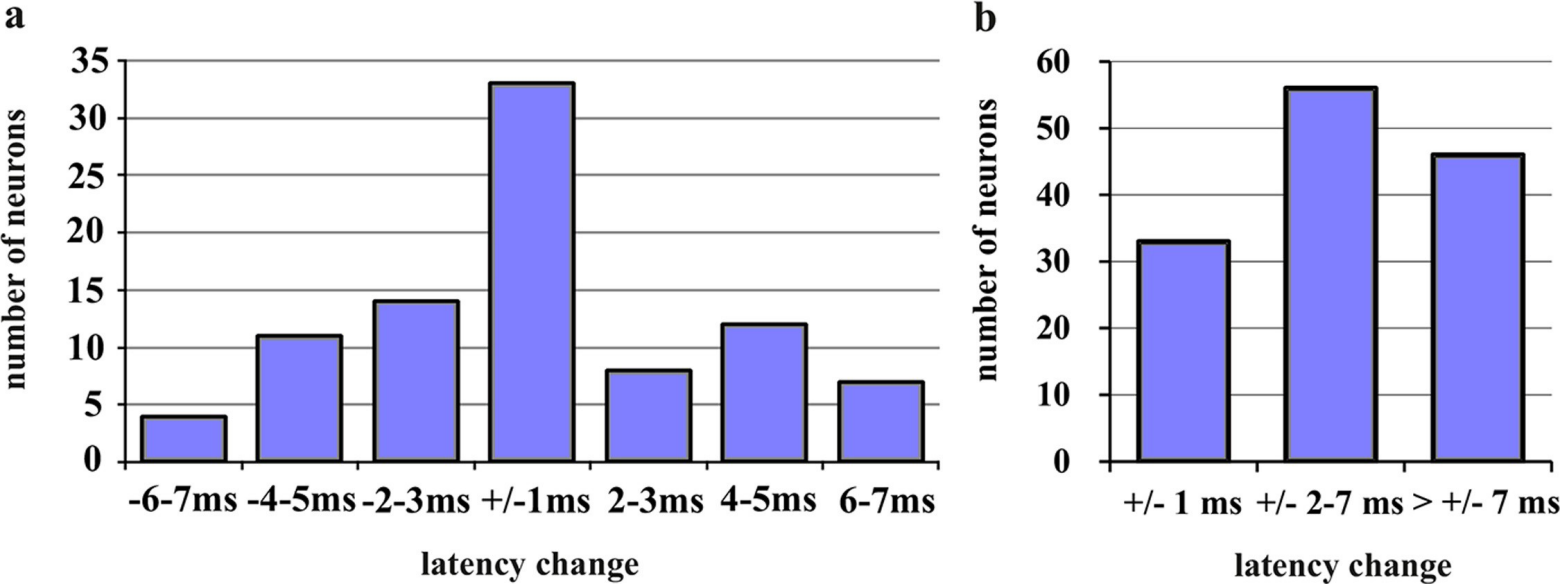
Numbers of neurons with different ranges of change of response latency within their FRAs. Ranges of latency change are with reference to response latency to CF tones at 30 dB above threshold. (a) Number of neurons with the indicated minor latency changes (from -6-7 ms up to 6–7 ms) in the core of their FRAs. (b) Number of neurons with the indicated latency changes in their whole FRAs. Latency changes > +/-7 ms occurred in neurons with *long-latency* responses.

The results of a detailed analysis of the variation of tone response latencies in the core part of the neurons’ FRAs are shown in Fig 11A. Plotted are the numbers of neurons with latency variations (latency at CF 30 dB above threshold as reference) of +/- 1 ms, - 2–3, - 4–5, - 6–7, + 2–3, + 4–5, and + 6–7 ms. Remarkably, a high number of neurons (33 = 24.4% of our sample) had very constant (only +/- 1 ms variation) tone response latencies in the core of their frequency response areas. Table 2 shows that most of them had class III FRAs with the shortest average latencies. Significant differences between the latency means were, however, not present (ANOVA, p > 0.1).

**Table 2:** Response latencies (ms) were averaged from neurons with constant (+/-1 ms) latencies in the core of their FRAs and plotted separately for the neurons in the 3 classes of FRAs.

Neuron class	Mean (ms)	SD (ms)	N
**Class I**	**13.0**	**1.2**	**5**
**Class II**	**14.1**	**3.2**	**9**
**Class III**	**10.9**	**2.1**	**19**
**all**	**12.1**	**2.7**	**33**

Means, standard deviations (SD) and number (N) of neurons in the classes are shown.

When we compare the number of the neurons with constant (+/-1 ms) latencies with the number of those of variable latencies (+/- 2–7 ms) in their core areas and with the other neurons with larger variations (> +/- 7 ms) in their whole FRAs (Fig 11B) and list all the neurons with regard to the shapes of their FRAs, we end up with the matrix of Table 3. Here, the distribution of the classes of latency variation in the classes of FRAs was highly significantly different (Chi^2^ = 36.15, 4 degrees of freedom, p < 0.001). The main differences came from the large numbers of class I neurons with latency variation within +/- 2–7 ms, of class II neurons with highly variable latencies, and of class III neurons with constant (+/- 1 ms) latencies.

**Table 3:** Distribution of neurons with different latency variation (Fig 11B) in the 3 classes of FRAs.

Neuron class	latency constant +/-1 ms	latency variable +/- 2–7 ms	latency highly variable > +/- 7 ms	all
**Class I**	**5**	**32**	**13**	**50**
**Class II**	**9**	**9**	**26**	**44**
**Class III**	**19**	**15**	**7**	**41**
**all**	**33**	**56**	**46**	**135**

In summary, the temporal responses throughout the FRAs of the neurons had strong relations to the shapes of the FRAs: More than the neurons in the other classes, class III neurons responded with purely *phasic* patterns of constant latencies. Many class II neurons had also *phasic* responses, however, with highly variable latencies. Class I neurons were least determined with their temporal response patterns, often showing variable latencies throughout their response areas. For neurons with *phasic*, *phasic-tonic*, *pauser* and/or *tonic* temporal response patterns, a core of the FRA could be defined. Within this core, the latency variation did not exceed +/- 7 ms with regard to the latency at the CF 30 dB above threshold. Larger latency changes (more than +/- 7 ms) always correlated with a change to the *long-latency* response or with the variability of response latency in neurons with only *long-latency* responses.

### Precision of tone-response latencies

**Fig 12:**
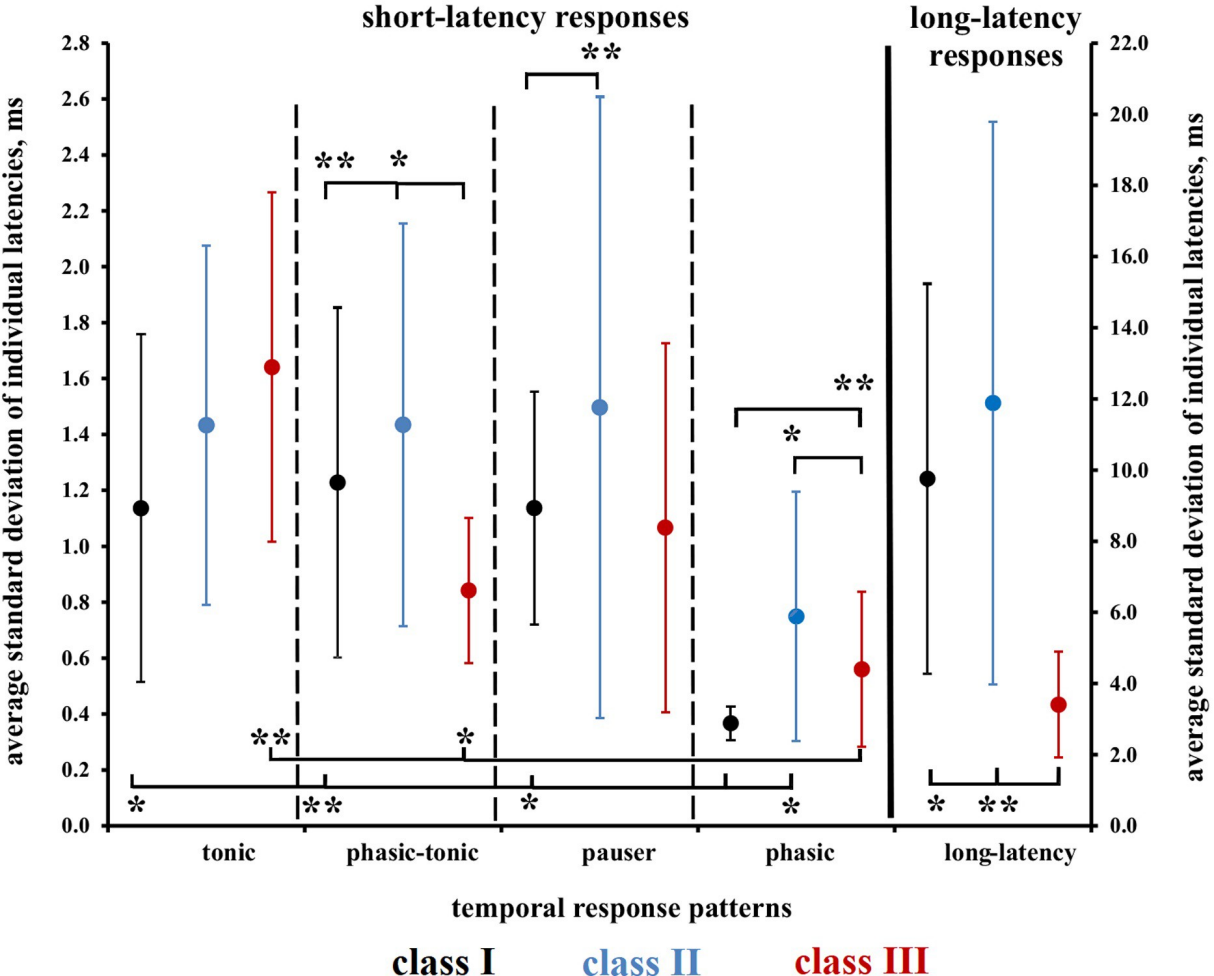
Latency jitter of individual neurons. Plotted are the means (with standard deviations) of the latency jitter of individual neurons with the indicated response patterns from the three classes of FRAs (black: class I, blue: class II, red: class III). The left y-axis refers to short-latency responses, the right y-axis to long-latency responses. Latency jitter equals the standard deviations of the tone-response latencies calculated from the 9 responses marked (red frame) in Fig 2. For further explanations, see text. Statistically significant differences between the means are indicated below the mean values, significant differences between the standard deviations above the mean values. * p < 0.05; ** p < 0.01.

The latency jitter of the first spike with which individual neurons responded to tone onset was evaluated with a resolution of +/- 10 μs. The standard deviation (SD) of 9 determinations of tone-onset response latencies per neuron (see Fig 2) served as measure for the amount of latency jitter of a given neuron. Thus, a small SD indicated small jitter or, in other words, high precision of tone-onset coding. The neurons included in our analysis showed only one of the temporal response patterns (*phasic*, *phasic-tonic*, *pauser*, *tonic*, or *long-latency*) in these 9 responses. Single latency outliers in 12 of 138 neurons responding with *phasic*, *phasic-tonic*, *pauser*, or *tonic* response patterns and in 15 of 40 neurons responding with *long-latency* response patterns have been excluded from the calculation of the SDs of latencies of the individual neurons. All samples of individual SDs leading to the average individual SD of the group of neurons with either *phasic*, *phasic-tonic*, *pauser*, *tonic*, or *long-latency* response patterns in each class of the FRAs were normally distributed. This justifies the calculation of the average SDs of individual neurons as indicators of the latency jitter or precision. The respective means (with SDs) of the individual SDs of *phasic*, *phasic-tonic*, *pauser*, *tonic*, or *long-latency* responding neurons in each class of the FRAs are plotted in Fig 12. The actual values and the corresponding means (with SDs and ranges), also of the group latencies are given in the S1 Table. The average tone-response latencies of neurons with different temporal response patterns (*phasic*, *phasic-tonic*, *pauser*, *tonic*, or *long-latency*) in the three classes of FRAs (S1A Table) were similar, often within the SDs of the samples, to the average latencies shown in Table 1. This suggests that the conditions of the latency measurements in the evaluated studies [23, 43, 54–56] were comparable.

Remarkably, the smallest latency jitter of individual neurons in our whole sample amounted to 130 μs and 170 μs occurring in neurons with *phasic* responses (classes II and III, S1B Table). In general, neurons with *phasic* responses were the most precise in their onset-response latencies with average individual SDs of 0.367 ms or 0.560 ms in classes I and III, respectively (Fig 12). Fig 12 also shows significant differences (indicated by stars below the mean values in the respective panels) in the precision of temporal responding between neurons of the 3 classes of FRAs and between neurons of different temporal response patterns within a given FRA class. Neurons with *phasic* responses in classes I and III had significantly smaller average individual SDs than neurons with *tonic*, *phasic-tonic*, and *pauser* (only class I neurons) responses (ANOVA on ranks followed by U-test). Further, class I neurons with *phasic* responses had smaller individual SDs than class II neurons of this response type, and class III neurons with *long-latency* responses had smaller individual SDs than class I and class II neurons (ANOVA followed by U-test in each case). The standard deviations of the means of the individual SDs are also shown in Fig 12. They indicated the variation of the latency jitter of the individual neurons among the analyzed neuronal populations. Class I and class III neurons responding with *phasic*, *phasic-tonic*, or *pauser* patterns had smaller standard deviations of the means than class II neurons with the respective response patterns. Significant differences (F-test) are indicated by stars above the mean values in the respective figure panels (Fig 12).

In summary, we found clear differences in latency jitter between neurons of the FRA classes and between temporal response types within a given class. On average, the tone-onset latencies of individual neurons with *phasic* responses were most precise, especially in neurons of FRA classes I and III. Compared to the neurons of the other FRA classes, class II neurons were least precise and the variation of the latency precision was largest among these class II neurons.

## Discussion

The importance of our results is threefold because they link together spectral and temporal analyses in the ICC of the mouse and couple both types of analysis with ICC space and with perception and perceptual constancy in the time domain. The following discussion will deal with these aspects.

### Temporal response patterns in FRAs of various shapes

The described temporal response patterns to tone bursts have been found associated with any shape of FRA (Fig 5 and Tables 1 and S1A) [21, 22, 25, 35, 44]. Our present data indicate, however, that neurons with class III FRAs responded preferentially and significantly more frequently with a short *phasic* spike burst to tone onset than the neurons in the other classes of FRAs (Figs 5 and 9). Actually, most of the 41 class III neurons showed constant *phasic* responses not only at their CF at 30 dB and higher above their response threshold (Figs 4 and 5) but also within their whole FRAs (Fig 9E; 23 = 56% of 41 neurons). Neurons of FRA classes I and II differed from class III in two respects: First, their temporal response patterns were much more variable (Fig 4) and intensity-dependent (Fig 6), i.e. only 14 (28% of 50) class I (Fig 9C) and 13 (29.5% of 44) class II neurons (Fig 9D) had constant temporal patterns in their FRAs compared to 24 (58.5%) of 41 class III neurons (Fig 9E). Second, if class I and II neurons had constant temporal response patterns in their FRAs at all, about 2/3 (class I) or 1/3 (class II) were not *phasic* but *tonic*, *pauser* or *long-latency* responses (Fig 9C and 9D). Thus, there were clear correlations both between constancy of temporal response patterns in the FRAs of ICC neurons and *phasic* response patterns (Fig 9B), and between these *phasic* patterns and class III FRAs (Fig 9E). The association of *phasic* response patterns with class III FRAs in the mouse ICC confirms previous results [44, 68]. They were gathered differently [44] via temporal response patterns from the whole FRA that had been summed up in a single PSTH resulting in virtually constant *phasic* responses in 47% of class III neurons.

Good candidates for the neurons with constantly *phasic* responses in their whole FRA are *phasic* or *transient* responders to depolarizing currents described in mouse and rat IC slices and *in vivo* studies [11–14, 69–73]. These neurons intrinsically responded, whenever activated above threshold, with one or few spikes at short latency. Since class III neurons were largely without inhibitory influences on the responses within the excitatory FRAs [23, 54, 74], this *phasic* response seemed not to be shaped by inhibition and, therefore, can be expected also when the class III neurons were stimulated by spectrally complex sounds, not only by bursts of pure tones as in the present study. Although, we have shown examples of the constancy of this *phasic* response to complex sounds in previous papers [54, 74], further studies should settle this question about the presence of a specialized population of ICC neurons with rather broad, inhibition-free, V-shaped frequency tuning and *phasic* responding (if responses were given at all) to any sounds. In contrast to our class III specialized *phasic* responders (more than 50% of the class III neurons with stable *phasic* responses in their whole FRAs, Fig 9A and 9E), *phasic* responses were observed at some tone intensities in neurons with variable temporal response patterns from all three classes (Figs 3H, 3I, 3K and S3 and S4). Such *phasic* responses as part of temporal response variation have been found also in studies in which neurons were pre-hyperpolarized or inhibitory input activated before or in addition to excitatory stimulation [10, 15, 62, 63, 71, 75, 76], or as part of sound duration tuning in the ICC [69, 76, 77]. Therefore, available evidence suggests that variable temporal response patterns of a neuron to tone bursts can be expected in presence of excitatory/inhibitory interactions within and around the neuron’s FRA (most of our class I and II neurons, few of our class III neurons) and, especially, if complex sounds with several frequency components or noise stimuli had to be processed [5, 10, 14, 20–22, 54].

### Latencies, latency precision, and response patterns in FRAs of various shapes

Besides tone-response patterns, tone-response latencies could vary within the FRAs of the neurons. Latencies of guinea pig, rat, bat, and cat ICC neurons with temporal response patterns such as *phasic*, *tonic*, *phasic-tonic*, *pauser* (and variants thereof) have been reported to be, on average, in a range of up to about 15 ms [39, 47, 69, 78, 79], in the mouse ICC up to about 20 ms [21, 80, 81], which was the value we used here to separate short-latency from long-latency responses (latencies > 20 ms). Average latencies of the short-latency response patterns (*phasic*, *tonic*, *phasic-tonic*, *pauser*) and of the neurons in the three classes of FRAs did not differ significantly (Tables 1 and S1A). However, the latency variation differed significantly among the neurons of the three FRA classes (Fig 11B and Table 3). Compared to the other classes, more class I neurons had responses with variable (+/- 2–7 ms) latencies, more class II neurons had responses with highly variable (> +/- 7 ms) latencies, and more class III neurons had responses that were least variable (+/- 1 ms). When we relate these differences in latency variation to the differences of variation of tone-response patterns between the neurons of the FRA classes (Figs 4, 5 and 9), we can state that, on average, class III neurons tended to have constant *phasic* responses with least latency variation within their FRAs. Neurons of classes I and II tended to change the temporal response patterns, dependent on sound-intensity at the CF (Fig 6), and within their whole FRAs (Fig 9A), coming along with considerable latency variation, especially when changes to long-latency responses occurred.

Latency variation concerns groups of neurons, latency precision concerns the amount of jitter in the latency of a single neuron in response to a number of sounds. Our results clearly showed that neurons of FRA classes I and III with *phasic* responses had both the smallest latency jitter and jitter variation compared with neurons of other temporal responses of the same FRA class and with neurons of FRA class II (Fig 12). The average values of first spike latency jitter of 0.367 ms (class I) and 0.560 ms (class III) were very similar to jitter values < 1 ms found in the ICC of the mouse [81], rat [82], cat [47], guinea pig [39], and bat [83, 84]. These values of latency jitter were in the range of 0.1–1 ms as found in the auditory nerve and auditory cortex [65]. Therefore, they support the notion [65] that the precision of sound-onset coding is constantly high and probably very similar at the levels of the auditory pathways from the cochlea to the auditory cortex. With our present data, we can modify this statement for the ICC by considering neurons with different shapes of FRAs. High precision of sound-onset coding in the ICC was, on average, present only in neurons of FRA classes I and III (Fig 12) whose FRAs were not dominated by inhibition as was the case for class II neurons [23, 54, 74].

Since in the studies on the ICC of other mammals latency jitter was neither related to shapes of FRAs nor to different temporal-response patterns, we can add two further aspects on the basis of our present results on mice: (1) Coding of sound onsets with highest precision (smallest jitter) in the ICC is, on average, the mission of neurons with short-latency *phasic* responses (Fig 12). Very similar, neurons from the ventral nucleus of the lateral lemniscus of bat [85] and cat [86] with *phasic* responses showed, on average, significantly smaller latency jitter (< 1 ms) than neurons with sustained (*tonic* and *phasic-tonic*) responses (> 1ms up to 10 ms in cat). In conclusion, neurons with sustained, especially *tonic* or *phasic-tonic* responses in the ventral lateral lemniscus and ICC may not provide the information decisive in onset-time-critical auditory perceptual tasks. Gap detection is such a task with detection thresholds of 2–3 ms gap duration in chinchillas and mice [87, 88]. Neurons with *phasic* responses in the ICC of the mouse were sensitive to gap durations in noise of 1–2 ms, neurons with *sustained* responses needed gap durations of about 4 ms for detection [80, 88]. Thus, *phasic* responses of ICC neurons, especially with FRAs of classes I and III with smallest single-unit latency jitter and smallest jitter variation among neurons (Fig 12), seem to be the basis for the perception of gaps in noise. (2) Neurons with *long-latency* responses (Figs 1D and S6) or *long-latency* responses as result of changes of sound intensity or frequency in the FRA (S5 Fig) had latency jitters roughly tenfold as large as those of the short-latency responses (Fig 12). Such neurons were unsuitable for coding in the time domain unless high precision was not a decisive factor as in discrimination of sound duration. For durations longer than about 30 ms, a latency jitter of 3–4 ms as observed in class III neurons (Fig 12) is at or below the discrimination threshold in humans [89] and far below that of mice [90]. Therefore, *long-latency* neurons, especially those of FRA class III with comparably small latency jitter (Figs 12 and S6A) might be involved in sound-duration coding in the ICC [91].

### Spatial gradients in the ICC present evidence for optimal coding locally, either in the spectral or temporal domain

The present results give reason to put labels from temporal response characteristics analyzed here to the neurons characterized before with regard to responses in the spectral domain (properties and shapes of FRAs) and to their distribution in the ICC space. In the ICC space of the mouse, class I neurons have been found everywhere, in all frequency-band laminae studied (12–32 kHz) with rather even distribution, class II neurons were concentrated, with few exceptions, right in the center of frequency-band laminae and in medial parts, and class III neurons were widely distributed in frequency-band laminae sparing the very center and becoming more frequent in the medial and lateral parts [43, 44].

In consequence, in the medial and lateral parts of the ICC many neurons could be expected to show short-latency *phasic* responses with high precision of the first-spike latency and high constancy of this response pattern in their whole FRAs, while *phasic* responses should be rare right in the center of a frequency-band lamina where onset responses with longer latency or of the *long-latency* type might be present. In fact, several studies indicated preferred places of onset (*phasic*) neurons in the medial and lateral ICC in the mouse [11], of onset neurons with shortest first-spike latencies and smallest latency jitter in the ventrolateral ICC of the gerbil [92], of onset-emphasized neurons with shortest first-spike latencies and smallest latency jitter in the rostrolateral ICC of guinea pigs [39], and of neurons with the shortest latencies in the lateral ICC of cats [36] and rostrolaterally in the ICC of chinchillas [38]. All these studies suggest that lateral parts of the ICC are specialized for sound-onset coding with short latencies of high precision of the first spike. Specifically, *phasic* neurons in the lateral ICC support superior responding to high-frequency amplitude modulations (repetition pitch) [36, 38] and fast frequency sweeps [44], and mapping of interaural time differences [93, 94] as basis for representation of sound azimuth angles (dorsolateral ICC [95]). On the other hand, the very center of the ICC showing comparably sharp frequency tuning [36, 41–43] with preferred locations of class II neurons [44] appears to be well adapted for coding in the spectral domain, however unsuitable for precise coding in the time domain.

In conclusion, the above-mentioned mapping data about neuronal response properties in the ICC support the notion that auditory brainstem input is subject to some transformation in the ICC with enhancement of spectral resolution by degradation of timing precision and enhancement of timing precision by degradation of spectral resolution [46, 47]. Neurons of FRA classes II and III appear to be representatives of such transformations, also with regard to transformation of auditory coding, by various aspects of neuronal responses, to certain local conditions in the ICC space [5, 32, 45]. The global relationship between first-spike latencies and sharpness of tuning (S7A and S7B Fig) and the correlations of short latencies with broad tuning in class III neurons and longer latencies with narrow tuning in class II neurons (Fig 7) appear like the general frame for functional and spatial organization of coding in the spectral and temporal domains in the ICC.

### The core of the neuronal FRA in relation to constancy in sound perception

The analysis of tone-response latencies in our present study revealed that neurons with short-latency responses (*phasic*, *tonic*, *phasic-tonic*, *pauser*) had a maximum of +/- 7 ms latency variation at their CF for tone levels of 30 dB and more above response threshold. By applying this window of latency variation to the whole FRA, we defined the core of the FRA as that frequency-intensity space in which a given ICC neuron (except those with *long-latency* responses) reliably responded to tone onsets. Reliability defined in this way means (5 ms rise time and 30 dB or more supra-threshold level of the tones considered) that the bulk of ICC neurons is expected to respond within about 15–20 ms [21, 39, 69, 78–81] after the time of tone onset. These 15–20 ms consider both the latency differences among the responding neurons (Fig 8) and the possible latency variation in single neurons due to changes of responses at various frequency-intensity combinations in their FRAs (up to 14 ms in the present study; Fig 11A). As proposed [96], the neural reference for the time of tone onset can be provided by neurons with constantly short and extremely precise first-spike latencies. According to our data (Figs 8, 9, 12), class I and class III neurons are the best candidates signaling sound onset as reference for and in the ICC, because they show short-latency *phasic* responses of highest precision. After this sharp start signal, the rest of the ICC neurons of the three classes that were activated by a given sound (except *long-latency* responders) join the sound-onset responding with their first spikes (several neurons also with their subsequent spikes after the first one) over about the next 14 ms when we consider 6 ms as the shortest first-spike latency in the ICC (Fig 8) under our present conditions (14 ms equals maximum 20 ms latency to tone onset minus 6 ms shortest response latency in the ICC). Thus, after about 14 ms all the sound-onset activation in the core of the FRAs of ICC neurons (except *long-latency* neurons) can be expected to have ceased and the ICC has become ready to respond newly either to the next sound onset in case of a sequence of transient sounds or to the next sharp amplitude increase in case of continuous sounds. Sounds with shallow (e.g. sinusoidal) amplitude modulations are not considered in the present context. In conclusion, the here defined core of the FRA of *single* ICC neurons represents a “window of reliability” of sound-onset responding in the frequency-intensity-time domains for the *whole* ICC with its “standard opening time” in the range of 14 ms.

In the Introduction, we mentioned speech intelligibility as example showing that perception and discrimination of auditory patterns improve with increasing sound levels and become rather constant at sound levels of about 30 dB above hearing threshold [48]. The perception of speech depends on the ability to detect sharp changes in sound amplitudes, characteristic of voice-onset-times of syllables starting with stop consonants, and of other consonant-vowel changes in fluent speech [97, 98], several of them involving sharp changes in sound amplitude. The shortest phonetic boundary on the continuum of voice-onset-time is that near 25 ms for /ba/ versus /pa/ discrimination both in humans [99, 100] and chinchillas [101]. Recordings from rat ICC neurons showed ability of consonant discrimination in speech if the neuronal spikes were evaluated with a temporal resolution better than about 20 ms [102]. Similarly, neurons in the ICC of chinchillas could reliably encode with their onset-response latencies voice-onset-times of synthetized vowels if the voice-onset-times were 20 ms and longer [103]. By adding the latency from tone onset to onset of activation of the ICC (6 ms under present conditions) with the 14 ms duration of the population onset-response (window of reliability) of the ICC neurons (except *long-latency* responders) we get to 20 ms which is in close correspondence to the shortest boundary of phoneme discrimination in speech based on voice-onset-time. A perceptual boundary near 20 ms time interval has also been found in other time-critical perceptual tasks in humans such as perception of the temporal order of sounds separated by a gap [104], sound integration across gaps in noise sequences [105], and perception of a sequence of auditory objects separated by gaps as a single stream or as two streams [106, 107]. Therefore, we hypothesize that the here defined core of FRAs of ICC neurons leading to an about 20 ms expected window of ICC responding with reference to sound onsets may be the neural source not only for setting the shortest boundary for voice-onset-time-based phoneme discrimination in speech but also as the shortest general perceptual boundary separating acoustically meaningful patterns by sound-onset time-domain. This general hypothesis for an ICC-based window of perceptual constancy of sounds in the time domain relies on how first-spike latencies to a sound are generated. Such latencies, as shown for auditory nerve fibers and neurons in the primary auditory cortex do not depend on the type of sound but only on the initial acceleration of the peak pressure [65, 66]. We assume that this also holds for neurons in the inferior colliculus. Then the core of the FRA of most neurons, as defined by the variation of first-spike latencies in our present study, will not depend on the type of sound (clicks, tones, noise, animal calls, speech, etc) as such but on the acceleration of the sound amplitude at the beginning of the sounds. This acceleration may naturally differ between sounds so that sounds with fast rise times of amplitudes such as clicks will lead to shorter latencies than, for example, the pronunciation of a vowel.

This hypothesis about the ICC origin of the shortest general boundary or time constant near 20 ms separating acoustically meaningful patterns by sound-onset time-domain cues may be tested for application in coding streams of animal calls by ICC neurons. An example are wriggling calls of mice, of which we know time-critical (within 20–30 ms) integration of formants in one stream for perception in unconditioned mother mice [108], and which are optimally processed by spectral facilitation of spike-rate responses in the ICC [54]. Another mouse example of constancy of perception in the time domain with a critical boundary near 20 ms is the case of categorical perception of ultrasonic tone bursts [109–111]. Mother mice divide a duration continuum of 50 or 60 kHz tones in non-preferred (durations ≤ 25 ms) and preferred (durations > 25 ms) sounds and discriminate between tones taken from the two categories. This labeling and discrimination of tone durations using a boundary near 20 ms happens in unconditioned mice suggesting, as in the case of wriggling-call perception (see above), that innate mechanisms of sound processing are responsible for setting the near 20 ms boundary [110–112]. Since unconditioned tone discrimination may not require auditory cortical processing [113], such discriminations could directly reflect sound processing in lower centers of the auditory pathways. The “window of reliability” of sound-onset responding in the ICC found in our present study may be part of the perceptually relevant subcortical processing assessing non-learned perceptual constancy in the temporal domain within the ICC. Similarly, perceptual constancy in the spectral domain via critical-band related perception has its counterpart in neuronal processing in the ICC [22, 31, 45, 54, 74, 112].

## Conclusions

The analysis of the excitatory frequency response areas (FRAs) of neurons in the central nucleus of the inferior colliculus (ICC) and the temporal response patterns to tone bursts in these FRAs showed partial interdependence of sharpness of tuning in the frequency (spectral) domain with constancy, precision, and promptness of responding to tone onsets in the time domain. This interdependence may also have correlates of partial spatial separation in the ICC. In addition, the variation of tone-response latencies in the FRAs of individual neurons gave rise, for most neurons, to the definition of a FRA core, which represents a time window of about 20 ms from tone onset for tone-onset responding of the whole ICC with possible relation to the roughly 20 ms time window found to be critical in several auditory perceptual tasks.

## Supporting information

S1 Fig Response analyses in the frequency and time domains: Two examples of neurons with class I frequency response areas (FRAs) and constant *tonic* (a) or *phasic* (b) responses. Upper left: Excitatory FRAs determined by three responses (spike bars) per frequency-intensity dyad with characteristic frequency (CF) indicated. Upper right: Matrix of average first-spike latencies in the corresponding FRA (left). Lower panels: Raster plots of spike responses to tones in a broad intensity range at the indicated frequencies (kHz) including the CF. Horizontal lines below the frequency specification indicate the tone duration (60 ms).
(TIF)

S2 Fig Response analyses in the frequency and time domains: Three examples of neurons with class II (a, b) or class III (c) frequency response areas and constant *phasic* responses. For further description, see S1 Fig.
(TIF)

S3 Fig Response analyses in the frequency and time domains: Two examples of neurons with class I (a) or class III (b) frequency response areas and variable *phasic-tonic/pauser/phasic* (a) or *phasic/pauser/phasic-tonic* responses (b). For further description, see S1 Fig.
(TIF)

S4 Fig Response analyses in the frequency and time domains: Two examples of neurons with class I (a) or class III (b) frequency response areas and variable *phasic/phasic-tonic/pauser* (a) or *phasic/pauser* (b) responses. For further description, see S1 Fig.
(TIF)

S5 Fig Response analyses in the frequency and time domains: Three examples of neurons with class I (a, b) or class III (c) frequency response areas and variable responses (a: *tonic/pauser/phasic/long-latency phasic*; b: *phasic/pauser/long-latency*; c: *phasic-tonic/pauser/long-latency*). For further description, see S1 Fig.
(TIF)

S6 Fig Response analyses in the frequency and time domains: Three examples of neurons with class III (a), class II (b) or class I (c) frequency response areas and mainly *long-latency* responses. For further description, see S1 Fig.
(TIF)

S7 Fig Relationship between the average tone-onset response latency in the whole FRA of a neuron and the width of its FRA expressed by Q_30_ (a) and Q_40_ (b) values. The shown linear regression lines indicate significant relations (see respective parameters on the plot).
(TIF)

S1 Table Evaluation of latency data of individual neurons for measuring the precision of responding by first-spike latency jitter. For each neuron, latencies were averaged from 9 responses to tones framed by the red box in Fig 2. **(a)** Latency data from the whole population of 178 studied neurons. First-spike latency means (ms) with standard deviations (SD), ranges of latencies (ms), and the number neurons (N) in the indicated response groups are shown. **(b**) Latency jitter was expressed by the standard deviations of the average latencies of individual neurons. These standard deviations were averaged across neurons and the means (ms), standard deviations of these means (ms), ranges of the means (ms), and the number neurons (N) in the different response groups are shown.
(DOCX)
