# Supplementary figures and images for: Frequency response areas of neurons in the mouse inferior colliculus. III. Time-domain responses: Constancy, dynamics, and precision in relation to spectral resolution, and perception in the time domain
Source: PLoS One. 2020 Oct 26;15(10):e0240853. doi: 10.1371/journal.pone.0240853 (PMC7588072; doi:10.1371/journal.pone.0240853)

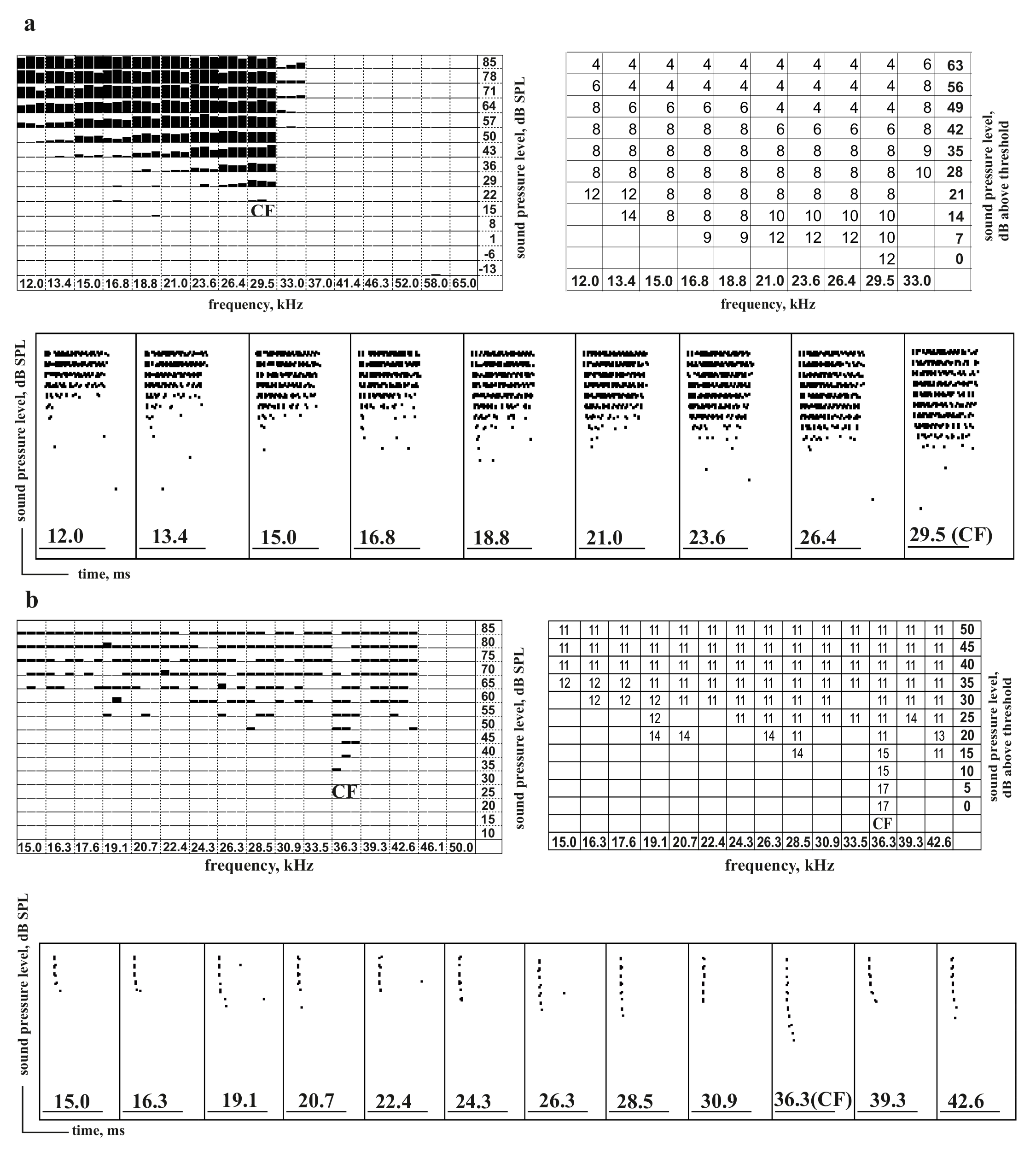

Supplement: S1 Fig — Response analyses in the frequency and time domains: Two examples of neurons with class I frequency response areas (FRAs) and constant tonic (a) or phasic (b) responses. Upper left: Excitatory FRAs determined by three responses (spike bars) per frequency-intensity dyad with characteristic frequency (CF) indicated. Upper right: Matrix of average first-spike latencies in the corresponding FRA (left). Lower panels: Raster plots of spike responses to tones in a broad intensity range at the indicated frequencies (kHz) including the CF. Horizontal lines below the frequency specification indicate the tone duration (60 ms). (TIF) [file pone.0240853.s001.tif]

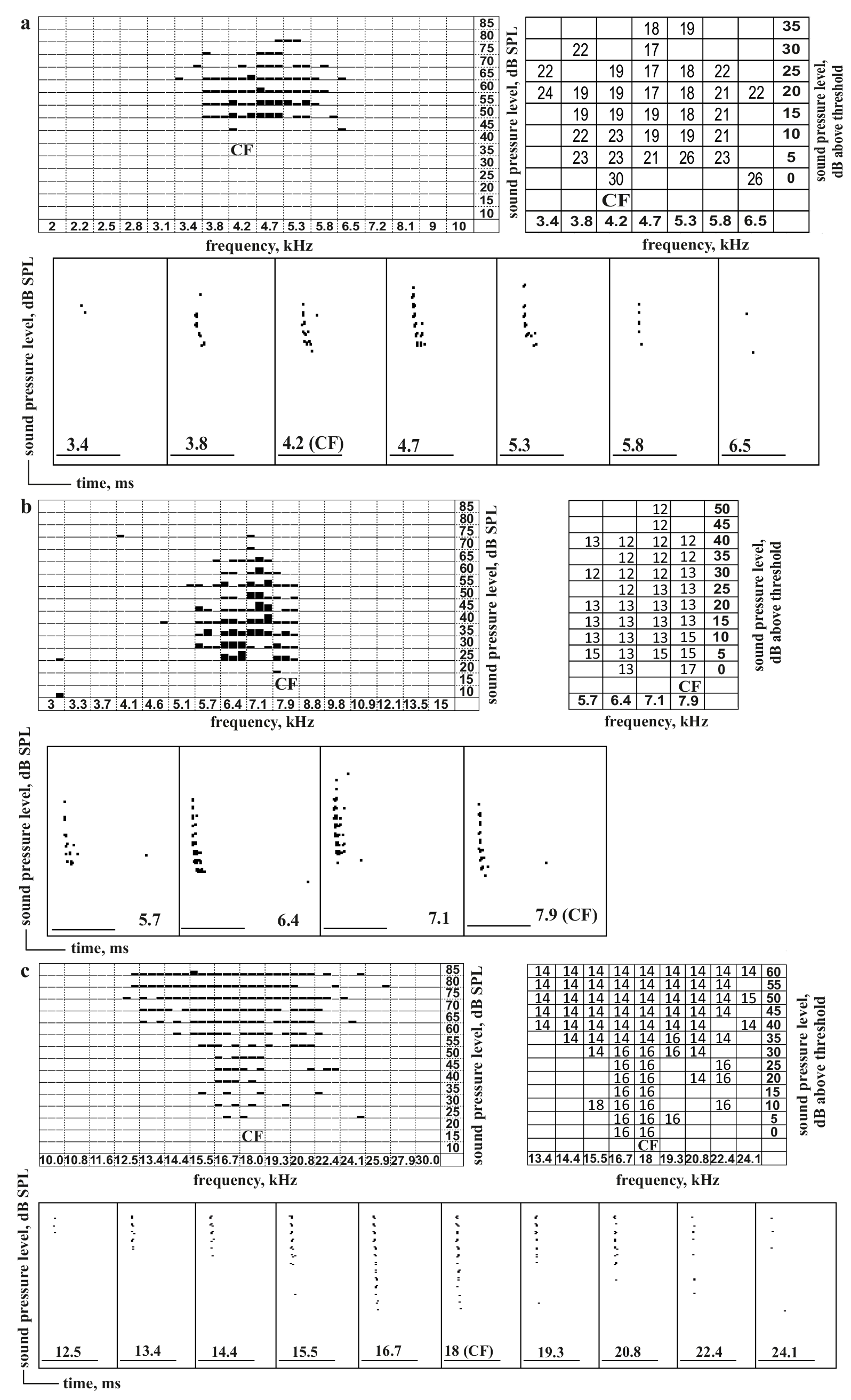

Supplement: S2 Fig — Response analyses in the frequency and time domains: Three examples of neurons with class II (a, b) or class III (c) frequency response areas and constant phasic responses. For further description, see S1 Fig. (TIF) [file pone.0240853.s002.tif]

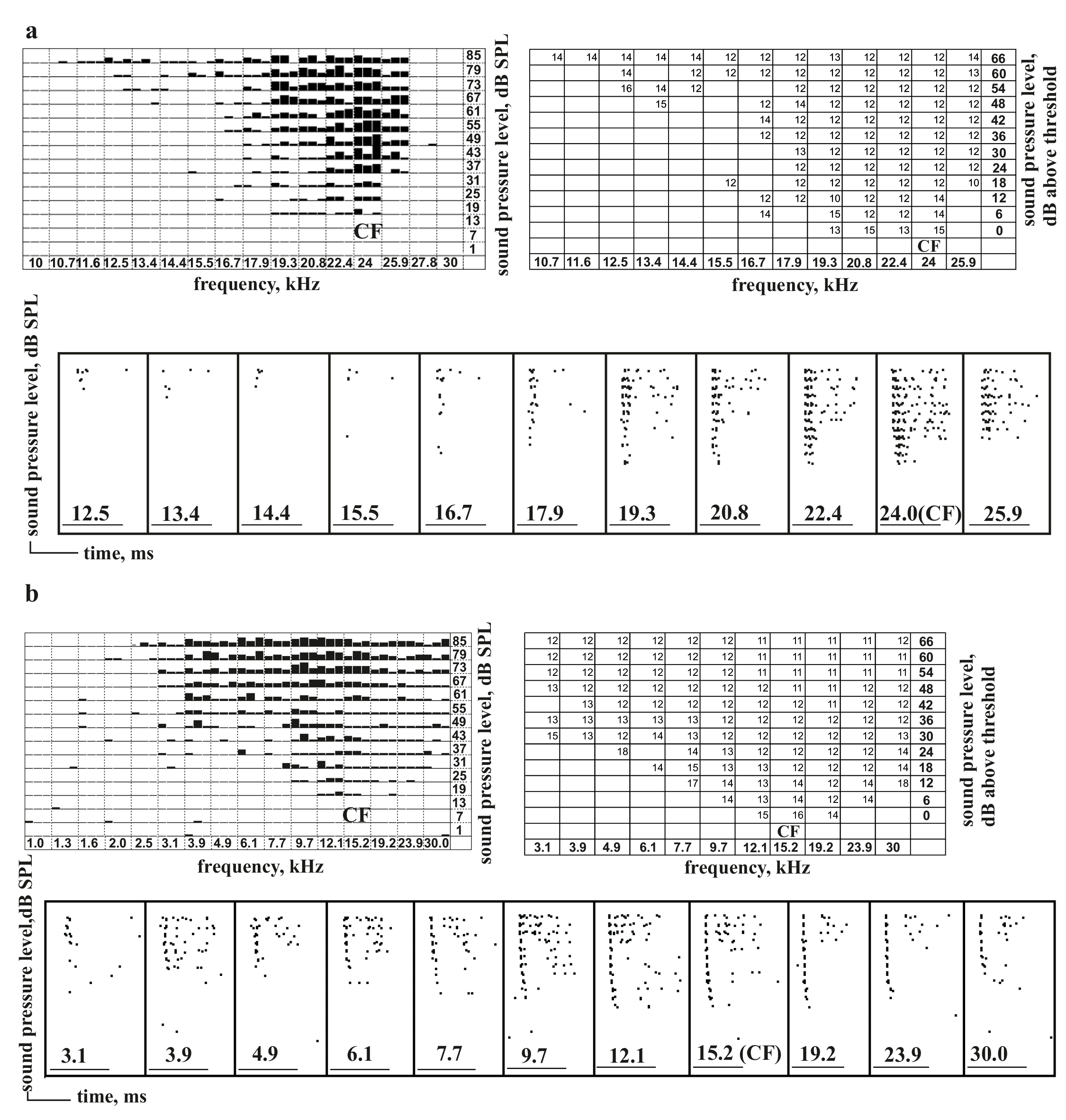

Supplement: S3 Fig — Response analyses in the frequency and time domains: Two examples of neurons with class I (a) or class III (b) frequency response areas and variable phasic-tonic/pauser/phasic (a) or phasic/pauser/phasic-tonic responses (b). For further description, see S1 Fig. (TIF) [file pone.0240853.s003.tif]

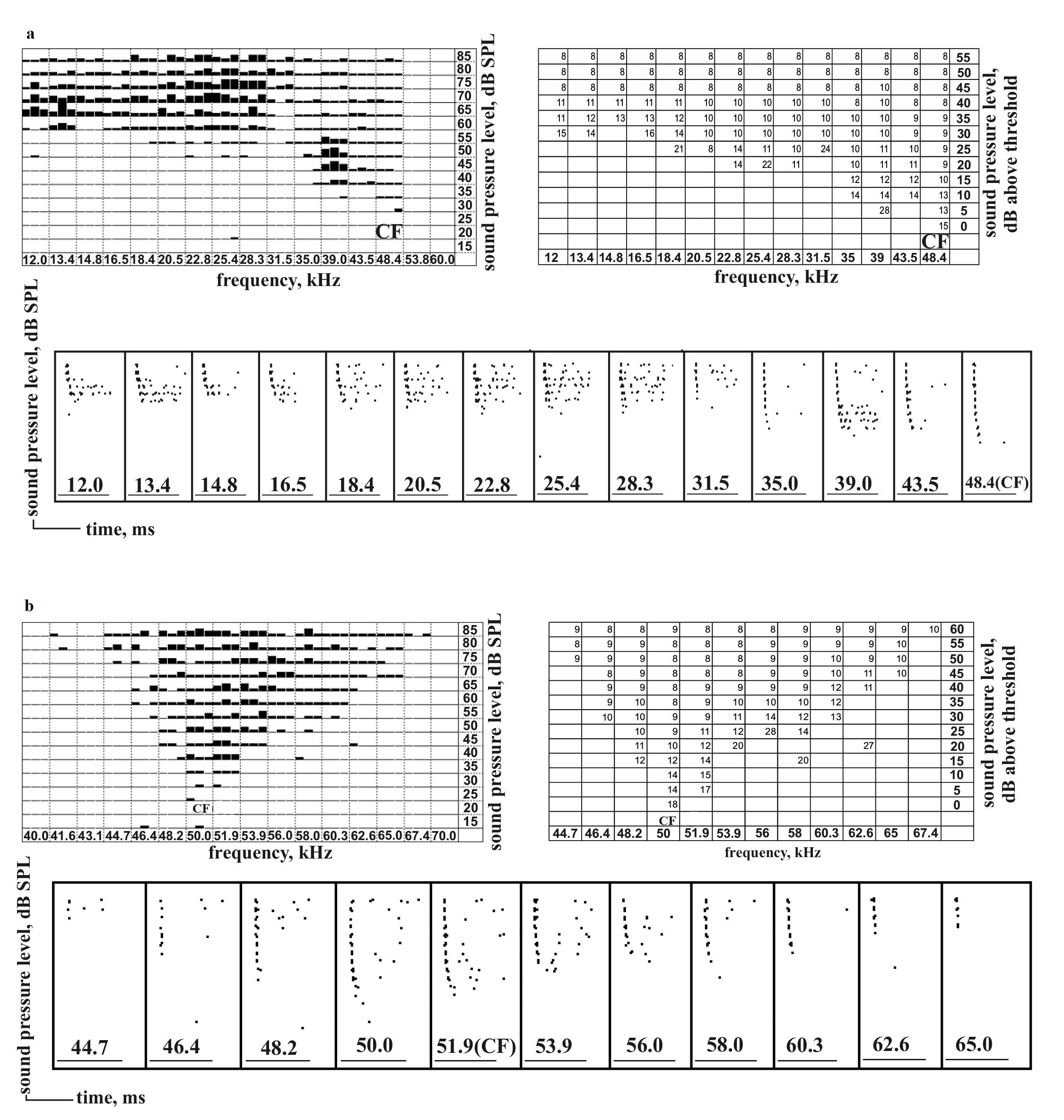

Supplement: S4 Fig — Response analyses in the frequency and time domains: Two examples of neurons with class I (a) or class III (b) frequency response areas and variable phasic/phasic-tonic/pauser (a) or phasic/pauser (b) responses. For further description, see S1 Fig. (TIF) [file pone.0240853.s004.tif]

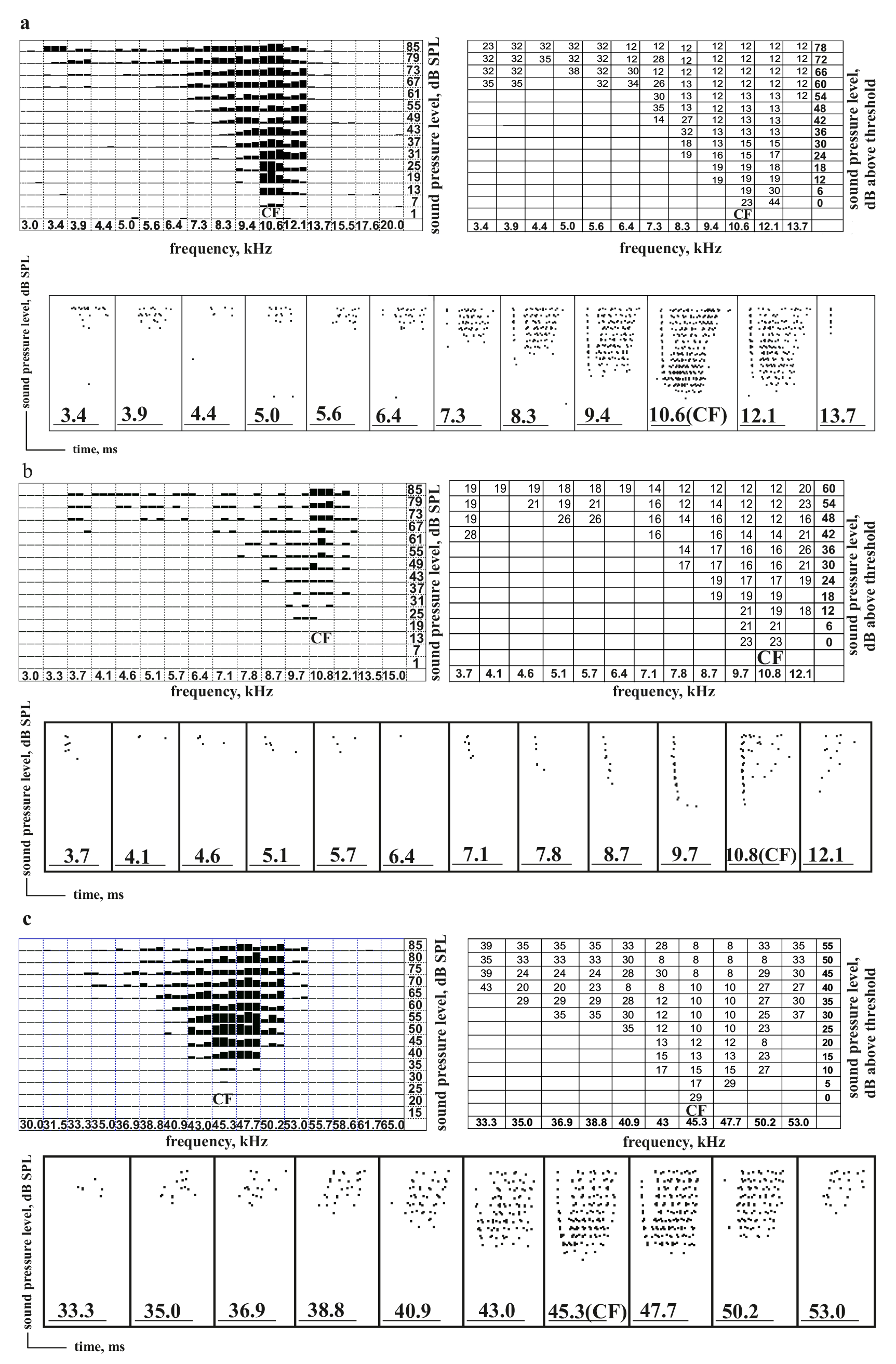

Supplement: S5 Fig — Response analyses in the frequency and time domains: Three examples of neurons with class I (a, b) or class III (c) frequency response areas and variable responses (a: tonic/pauser/phasic/long-latency phasic; b: phasic/pauser/long-latency; c: phasic-tonic/pauser/long-latency). For further description, see S1 Fig. (TIF) [file pone.0240853.s005.tif]

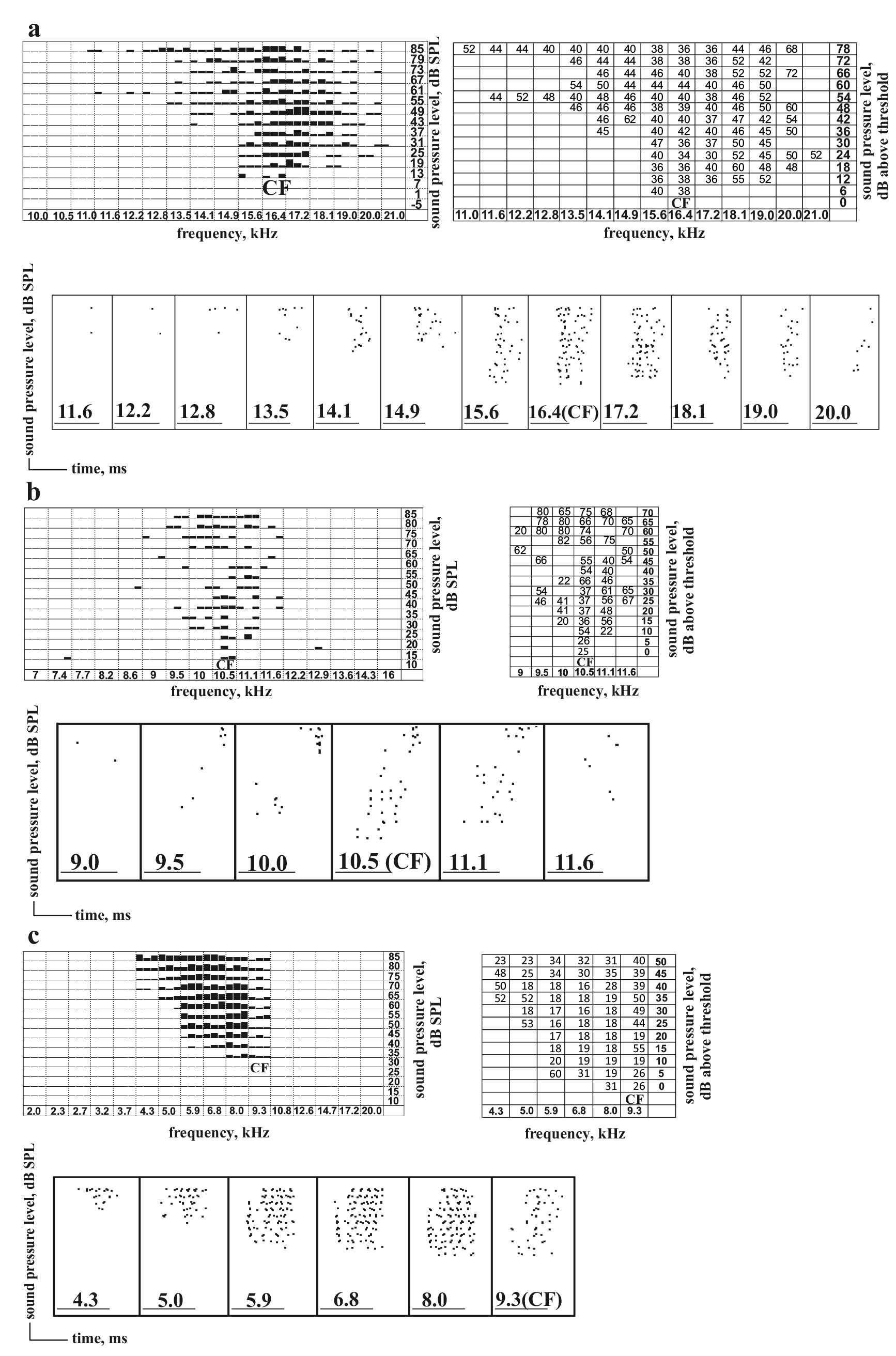

Supplement: S6 Fig — Response analyses in the frequency and time domains: Three examples of neurons with class III (a), class II (b) or class I (c) frequency response areas and mainly long-latency responses. For further description, see S1 Fig. (TIF) [file pone.0240853.s006.tif]

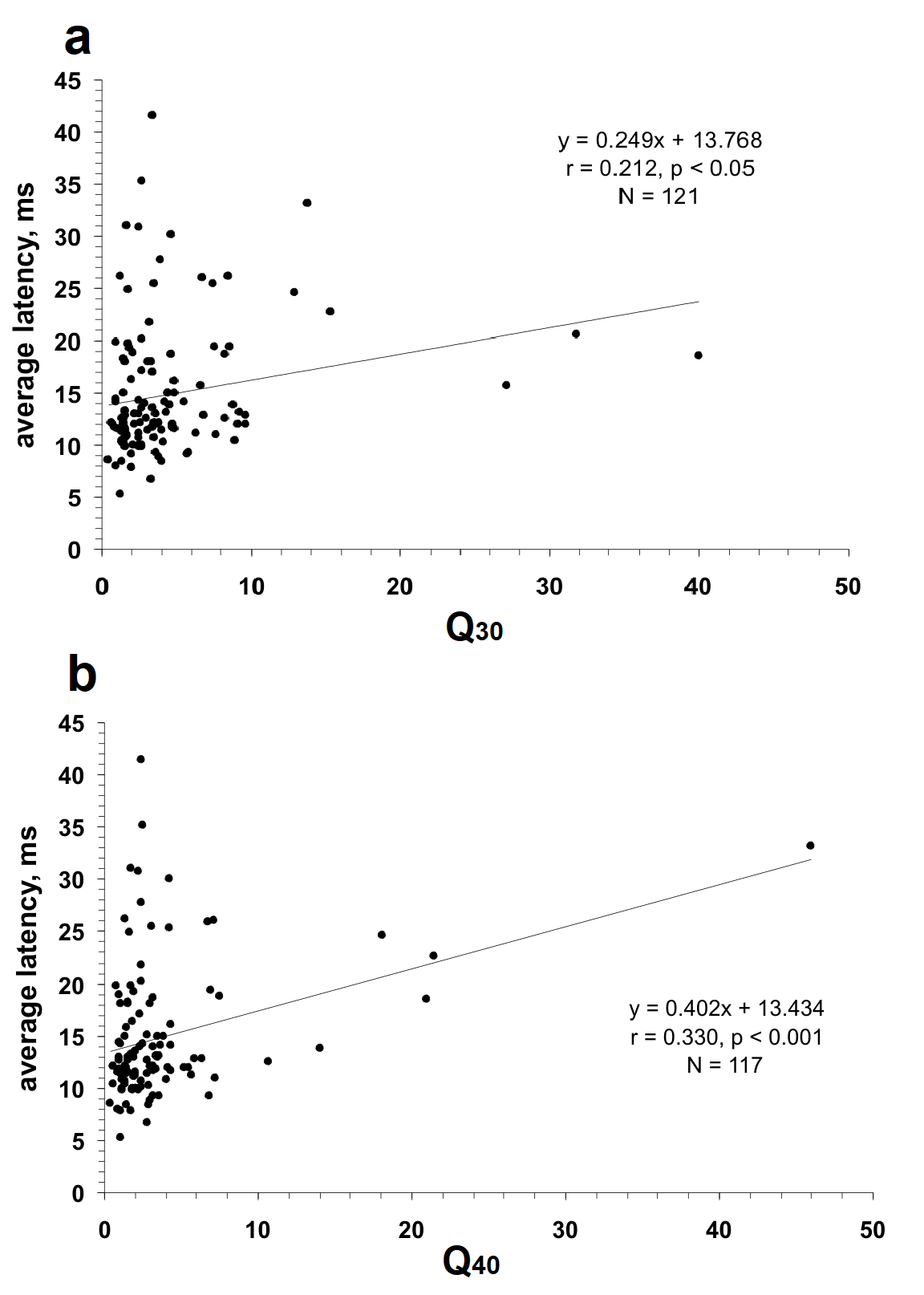

Supplement: S7 Fig — Relationship between the average tone-onset response latency in the whole FRA of a neuron and the width of its FRA expressed by Q30 (a) and Q40 (b) values. The shown linear regression lines indicate significant relations (see respective parameters on the plot). (TIF) [file pone.0240853.s007.tif]
