# Supplementary material for: Frequency response areas of neurons in the mouse inferior colliculus. III. Time-domain responses: Constancy, dynamics, and precision in relation to spectral resolution, and perception in the time domain
Source: PLoS One. 2020 Oct 26;15(10):e0240853. doi: 10.1371/journal.pone.0240853 (PMC7588072; doi:10.1371/journal.pone.0240853)
Supplement: S1 Table — For each neuron, latencies were averaged from 9 responses to tones framed by the red box in Fig 2. (a) Latency data from the whole population of 178 studied neurons. First-spike latency means (ms) with standard deviations (SD), ranges of latencies (ms), and the number neurons (N) in the indicated response groups are shown. (b) Latency jitter was expressed by the standard deviations of the average latencies of individual neurons. These standard deviations were averaged across neurons and the means (ms), standard deviations of these means (ms), ranges of the means (ms), and the number neurons (N) in the different response groups are shown. (DOCX) [file pone.0240853.s008.docx]

**S1 Table. Evaluation of latency data of individual neurons for measuring the precision of responding by first-spike latency jitter. For each neuron, latencies were averaged from 9 responses to tones framed by the red box in Fig 2.**

**Table a.** Latency data from the whole population of 178 studied neurons. First-spike latency means (ms) with standard deviations (SD), ranges of latencies (ms), and the number neurons (N) in the indicated response groups are shown.

| **Neuron class** | | **tonic** | **phasic-tonic** | **pauser** | **phasic** | **all short latency** | **long-latency** |
| --- | --- | --- | --- | --- | --- | --- | --- |
|  |  |  |  |  |  |  |  |
|  |  |  |  |  |  |  |  |
| **Class I** | **mean** | **11.572** | **11.388** | **12.100** | **12.720** | **11.726** | **36.738** |
|  | **SD** | **2.423** | **2.479** | **1.885** | **2.079** | **2.254** | **14.898** |
|  | **range** | **7.47 – 14.92** | **8.17 – 16.11** | **9.17 – 14.88** | **9.72 – 15.86** | **7.47 - 15.86** | **20.64 – 62.48** |
|  | **N** | **17** | **18** | **12** | **6** | **53** | **16** |
| **Class II** | **mean** | **13.241** | **11.861** | **12.332** | **11.401** | **11.791** | **33.708** |
|  | **SD** | **1.183** | **1.016** | **2.086** | **2.184** | **2.035** | **9.959** |
|  | **range** | **11.89 - 14.08** | **11.17 - 13.03** | **8.75 - 14.39** | **7.69 - 14.00** | **7.69 - 14.39** | **19.39 - 48.38** |
|  | **N** | **3** | **3** | **6** | **20** | **32** | **15** |
| **Class III** | **mean** | **13.171** | **13.610** | **12.771** | **12.740** | **13.013** | **24.553** |
|  | **SD** | **1.706** | **2.155** | **1.464** | **2.508** | **2.249** | **5.283** |
|  | **range** | **10.75 - 15.33** | **10.14 - 16.31** | **11.47 - 14.53** | **8.58 - 16.72** | **8.58 - 16.72** | **20.50 - 34.22** |
|  | **N** | **7** | **13** | **4** | **29** | **53** | **9** |
|  | **mean** | **12.661** | **12.286** | **12.401** | **12.287** | **12.236** | **31.666** |
| **all** | **SD** | **1.771** | **1.883** | **1.812** | **2.257** | **2.273** | **10.047** |
|  | **range** | **7.47 - 15.33** | **8.17 - 16.31** | **8.75 - 14.53** | **7.69 - 16.72** | **7.47 - 16.72** | **19.39 - 62.48** |
|  | **N** | **27** | **34** | **22** | **55** | **138** | **40** |

**Table b.** Latency jitter was expressed by the standard deviations of the average latencies of individual neurons. These standard deviations were averaged across neurons and the means (ms), standard deviations of these means (ms), ranges of the means (ms), and the number neurons (N) in the indicated response groups are shown.

| **Neuron class** | | **tonic** | **phasic-tonic** | **pauser** | **phasic** | **all short latency** | **long-latency** |
| --- | --- | --- | --- | --- | --- | --- | --- |
|  |  |  |  |  |  |  |  |
|  |  |  |  |  |  |  |  |
| **Class I** | **mean** | **1.136** | **1.228** | **0.848** | **0.367** | **1.015** | **9.757** |
|  | **SD** | **0.622** | **0.626** | **0.417** | **0.060** | **0.600** | **5.480** |
|  | **range** | **0.26 – 2.59** | **0.31 – 2.44** | **0.33 – 1.83** | **0.25 – 0.42** | **0.25 - 2.59** | **2.27 – 22.69** |
|  | **N** | **17** | **18** | **12** | **6** | **53** | **16** |
| **Class II** | **mean** | **1.433** | **1.435** | **1.497** | **0.749** | **1.017** | **11.887** |
|  | **SD** | **0.643** | **0.720** | **1.111** | **0.446** | **0.711** | **7.908** |
|  | **range** | **0.91 - 2.15** | **0.72 - 2.16** | **0.22 - 2.90** | **0.17 - 1.96** | **0.17 - 2.90** | **2.47 - 28.97** |
|  | **N** | **3** | **3** | **6** | **20** | **32** | **15** |
| **Class III** | **mean** | **1.641** | **0.842** | **1.067** | **0.560** | **0.810** | **3.406** |
|  | **SD** | **0.625** | **0.260** | **0.661** | **0.278** | **0.511** | **1.484** |
|  | **range** | **0.99 - 2.60** | **0.52 - 1.25** | **0.39 - 1.88** | **0.13 - 1.19** | **0.13 - 2.60** | **1.23 - 5.23** |
|  | **N** | **7** | **13** | **4** | **29** | **53** | **9** |
|  | **mean** | **1.400** | **1.168** | **1.137** | **0.559** | **0.937** | **8.350** |
| **all** | **SD** | **0.630** | **0.535** | **0.730** | **0.261** | **0.600** | **4.957** |
|  | **range** | **0.26 - 2.60** | **0.31 - 2.44** | **0.22 - 2.90** | **0.13 - 1.96** | **0.13 - 2.90** | **1.23 - 28.97** |
|  | **N** | **27** | **34** | **22** | **55** | **138** | **40** |
